# Comprehensive profiling of alternative splicing landscape during cold acclimation in tea plant

**DOI:** 10.1186/s12864-020-6491-6

**Published:** 2020-01-20

**Authors:** Yeyun Li, Xiaozeng Mi, Shiqi Zhao, Junyan Zhu, Rui Guo, Xiaobo Xia, Lu Liu, Shengrui Liu, Chaoling Wei

**Affiliations:** State Key Laboratory of Tea Plant Biology and Utilization, Anhui Agricultural University, West 130 Changjiang Road, Hefei, Anhui 230036 People’s Republic of China

**Keywords:** AS isoforms, *Camellia sinensis*, Cold adaptation, Cold stress, Transcriptome

## Abstract

**Background:**

Alternative splicing (AS) may generate multiple mRNA splicing isoforms from a single mRNA precursor using different splicing sites, leading to enhanced diversity of transcripts and proteins. AS has been implicated in cold acclimation by affecting gene expression in various ways, yet little information is known about how AS influences cold responses in tea plant (*Camellia sinensis*).

**Results:**

In this study, the AS transcriptional landscape was characterized in the tea plant genome using high-throughput RNA-seq during cold acclimation. We found that more than 41% (14,103) of genes underwent AS events. We summarize the possible existence of 11 types of AS events, including the four common types of intron retention (IR), exon skipping (ES), alternative 5′ splice site (A5SS), and alternative 3′ splice site (A3SS); of these, IR was the major type in all samples. The number of AS events increased rapidly during cold treatment, but decreased significantly following de-acclimation (DA). It is notable that the number of differential AS genes gradually increased during cold acclimation, and these genes were enriched in pathways relating to oxidoreductase activity and sugar metabolism during acclimation and de-acclimation. Remarkably, the AS isoforms of *bHLH* transcription factors showed higher expression levels than their full-length ones during cold acclimation. Interestingly, the expression pattern of some AS transcripts of raffinose and sucrose synthase genes were significantly correlated with sugar contents.

**Conclusion:**

Our findings demonstrated that changes in AS numbers and transcript expression may contribute to rapid changes in gene expression and metabolite profile during cold acclimation, suggesting that AS events play an important regulatory role in response to cold acclimation in tea plant.

## Background

Low temperature is one of the most important environmental factors affecting plant growth, development and geographical distribution. Cold acclimation (CA) is an important mechanism that has been widely reported to improve cold resistance of plants by modulating numerous physiological and biochemical processes [1–3]. For instance, cold acclimation improves the tolerance of North American Rhododendron from − 7 °C to − 53 °C [4]. During CA, the contents of soluble sugars including sucrose, fructose, glucose and raffinose increase significantly, and these sugars are thought to osmotically stabilize membranes [5, 6]. Unsaturated fatty acids also play an important role in cold resistance, which has previously been demonstrated in tobacco [7]. In addition to increases of these substances, many transcription factors and oxidoreductase regulatory mechanisms are also present in plants during cold acclimation. For example, The *CBF* (C-repeat-binding factor) transcription factor regulates *COR* (cold-regulated) genes which function in the response to low temperature [8]. Plants can also defend against active oxygen through the protection system of oxidoreductases during cold acclimation [9]. Cold acclimation grants the ability to withstand low temperature and plays an important role in the growth and development of plants.

Alternative splicing (AS) is the process by which different mRNA splicing isoforms are produced from a single mRNA precursor by different splicing methods.AS can generate multiple transcript and protein isoforms from the same gene [10, 11]. Evidence is accumulating that AS plays a crucial role in a variety of plant development processes and stress responses, including regulation of flowering [12], defense response to pathogenic bacteria [13], and abiotic stress response [14, 15]. The targets of AS in rice were found to include three Serine/arginine-rich (SR) protein-encoding genes regulating phosphorus (P) uptake and remobilization in a highly nutrient-specific manner, thus demonstrating that AS plays a critical role in maintaining mineral nutrient homeostasis [16]. Previous studies have reported that temperature-associated alternative splicing is an important mechanism involved in the regulation of flowering and the plant circadian clock [12, 17]. Under cold stress, hundreds of genes in *A.thaliana* showed transcriptional changes due to rapidly occurring AS and AS can affect several cold-responsive transcription factors and RNA binding proteins [18, 19]*.* In durum wheat, low temperature promotes intron retention in two early cold-regulated genes [20]. In temperature signaling cascades in plants, AS is considered as a way of perceiving temperature fluctuations and modulating transcription factor activity, perhaps by linking regulation of gene expression with PEPi (Peptide interference) and/or NMD (Nonsense-mediated decay) mechanisms [21]. However, most of these studies focus on the low temperature, and few address on plant cold acclimation, especially the AS changes after de-acclimation.

The tea plant (*Camellia sinensis*) is a perennial evergreen woody crop and its leaves are used for making tea beverage, which is one of the three popular nonalcoholic beverages consumed worldwide [22, 23]. It is popular among most consumers due to its good taste and health-promoting effects [24, 25]. Tea plants are vulnerable to low temperatures in winter, especially in northern China [26]. In tea plant, the contents of sucrose, glucose and fructose were found to be constantly elevated during cold acclimation [27]. In addition, significantly increased expression of *CBF* (C-repeat-binding factor) and *DHN* (dehydrin) occurs during cold acclimation [28]. Both increases in sugar contents and expression of related genes can improve the cold resistance in tea plant. But these studies have not focused on the effect of AS on cold stress in tea plants. In our previous study, we found that some AS events were tissue specific in stem and root; we also found that some AS isoforms were the major transcripts involved in the flavonoid synthesis pathway, which suggested AS is positively correlated with the contents of catechins [29]. We also reported that six *CsLOXs* (lipoxygenase) varied significantly in relative abundances under the different stresses [30]. However, these studies on AS emphasize largely on secondary metabolism.

In this study, we investigated AS events during cold acclimation with genome-wide analysis in tea plant, which detected a large number of AS occurrences. Meanwhile, variations of AS and the related biological functions were analyzed during cold acclimation. Our results indicated that AS may regulate gene expression and contents of metabolites during cold acclimation. A number of different AS patterns were found being involved in transcriptional regulation during the process of cold acclimation in tea plants, especially the change and function of AS at de-acclimation. This provides a better understanding of the functions of AS in tea plants responding to cold acclimation.

## Results

### Global identification and classification of AS events

**Fig. 1:**
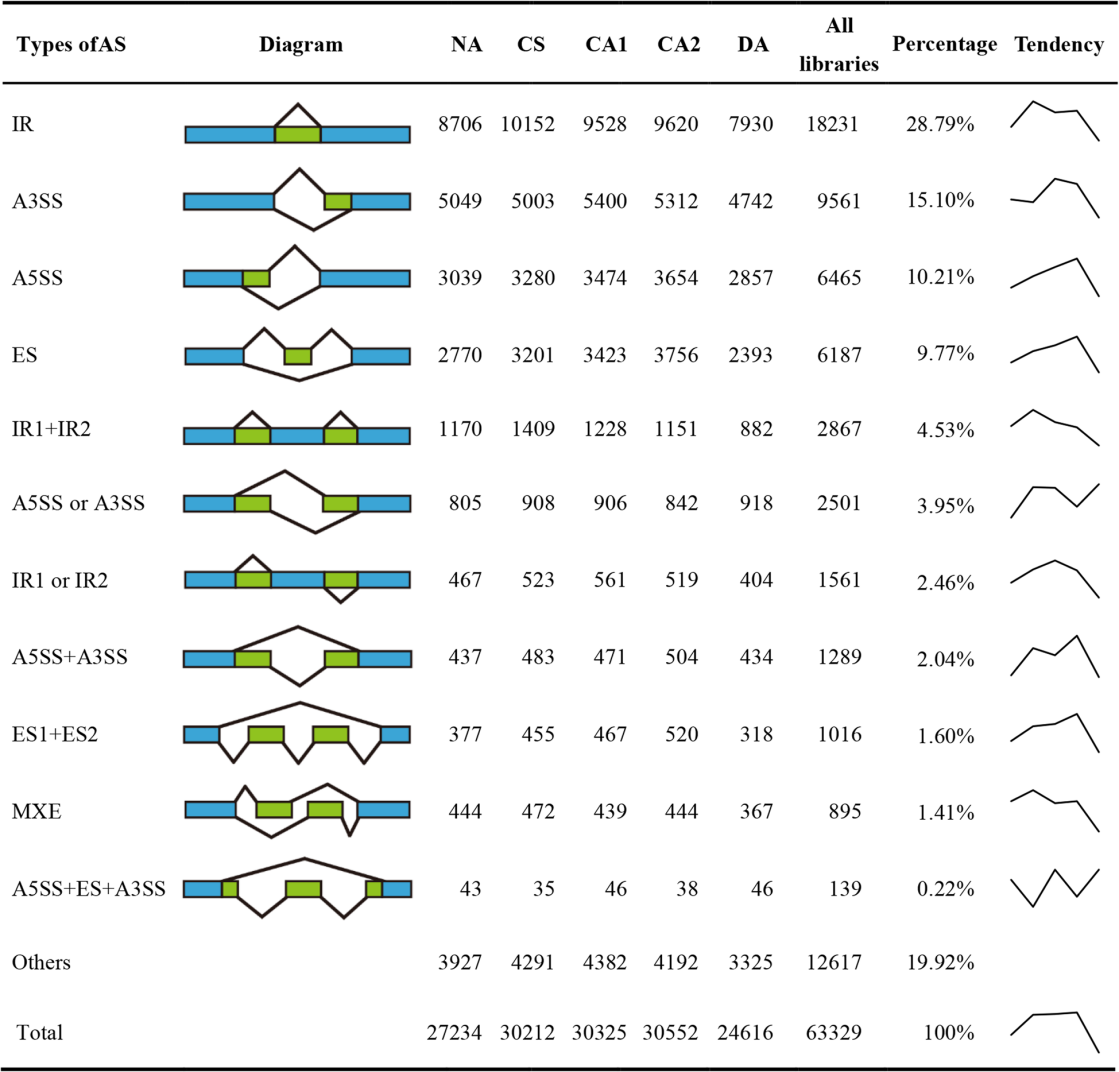
Statistics of all AS events at different time points of cold acclimation treatments. IR: intron retention; ES: exon skipping; A3SS: alternative 3′splice site; A5SS: alternative 5′ splice site; MXE: mutually exclusive exon. NA: non-acclimation; CS: cold stress of 6 h at 10 °C, day/night; CA1: cold acclimation of 7 days at 10/4 °C, day/night; CA2: cold acclimation of 7 days at 4/0 °C, day/night; DA: de-acclimation of 7 days at 25/20 °C, day/night. The tendency represents the changes in the number of AS events at cold acclimation time points

The RNA-seq data was used to investigate AS events at different periods of cold acclimation (Additional file 4: Table S4). Briefly, clean reads were initially mapped to the tea plant reference genome, and AS events were then identified using the AStalavista tool. From this analysis, a total of 63,329 AS events were identified from 14,103 genes in all samples (Fig. 1). Among them, IR was the most abundant type (18,231, 28.79%), followed by A3SS (9561, 15.10%), A5SS (6465, 10.21%) and ES (6187, 9.77%) (Fig. 1). In addition, a large number of other types of AS were detected in all samples because of multiple splicing modes occurring on a single transcript. These results were consistent with those of previous reports in other plant species [31]. During the sharp cooling treatment of tea plants, the numbers of AS events under low-temperatures [cold acclimation of 6 h at 10 °C, day/night temperature (CS), cold acclimation of 7 days at 10/4 °C, day/night temperature (CA1) and cold acclimation of 7 days at 4/0 °C, day/night temperature (CA2)] were significantly increased relative to AS events in those growing under normal temperature (NA, 25/20 °C, day/night temperature); thus, the amount of CS (30,212), CA1 (30,325) and CA2 (30,552) was about 3000 more than NA (27,234), respectively. It is noteworthy that the number of AS events (24,616) tremendously decreased under temperature recovery condition compared with both CA treatment and NA groups, implying that many AS genes had adapted to low-temperature environment and resulted in reduced occurrence of AS events during DA (de-acclimation) of tea plant.

**Fig. 2:**
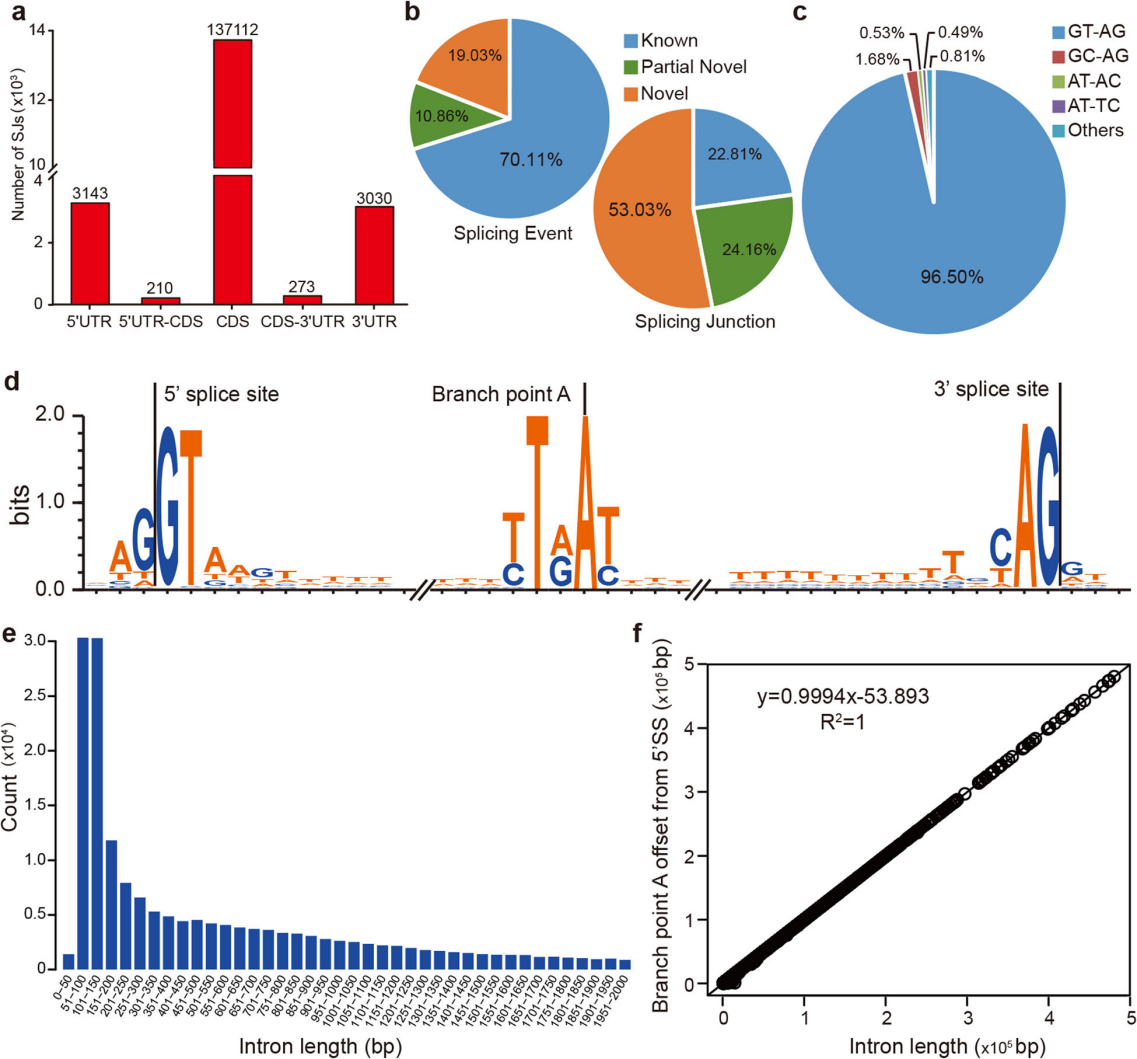
Analysis of splice junctions. **a** Distribution of splicing junctions in the annotated loci. **b** Distribution of splice events and splice junction levels. **c** Percentages of splicing donor-acceptor di-nucleotide usages among all transcripts. **d** Sequence logos of intronic 5′ splice sites, branch sites, and 3′ splice sites; logos were created using WebLogo 3. **e** Distribution of lengths of introns based on RNA-Seq (lengths of introns ≤2000 bp). **f** Relationship between branch point A offset from 5′splice site and intron length. Splice junction: multiple splicing events spanning the same intron were considered as one splicing junction. Annotated (known): The junction is part of the gene model. Both splice sites, 5′ splice site (5′SS) and 3′splice site (3’SS) are annotated by reference gene model. Complete_novel: Both 5′SS and 3′SS are novel. Partial_novel: One of the splice site (5′SS or 3′SS) is novel, and the other splice site is annotated

Considering the isoforms in the annotated loci, most of the splicing junctions (SJs) were found to reside in the coding regions (or coding sequence) (CDS) (137,112), while fewer SJs were observed in 5′ untranslated region (5′UTR) (3143) and 3′ untranslated region (3′UTR) (3030), highlighting that coding proteins were intensively influenced by AS (Fig. 2a). At the splice event level, the number of known splice annotation was the highest followed by partial novel. Whereas, the novel splice annotation accounted for the greatest number at the splice junction level (Fig. 2b). With regard to the splicing donor-acceptor sites, a majority (96.50%) were found to be the canonical GU-AG, followed by GC-AG with only 1.68% (Fig. 2c and Additional file 1: Table S1). The noncanonical AU-AC splice site pair, specifically (U12-type introns) are thought to have important regulatory roles [32], and accounted for only 0.53% (Fig. 2c). The 5′ss and 3′ss sequences are shown in Fig. 2d. We located the branch site and branch point A for almost all of the SJs (Fig. 2e). The average distance from the branch point A to 3′ss was 54.5 ± 86.6 bp in length. Most introns were found to be 51 bp - 150 bp in length, the average intron length was 2527.04 ± 12,234.5 bp, and the median was 500 bp. The branch point A offset from the 5’ss was positively correlated with intron length (Fig. 2f).

**Fig. 3:**
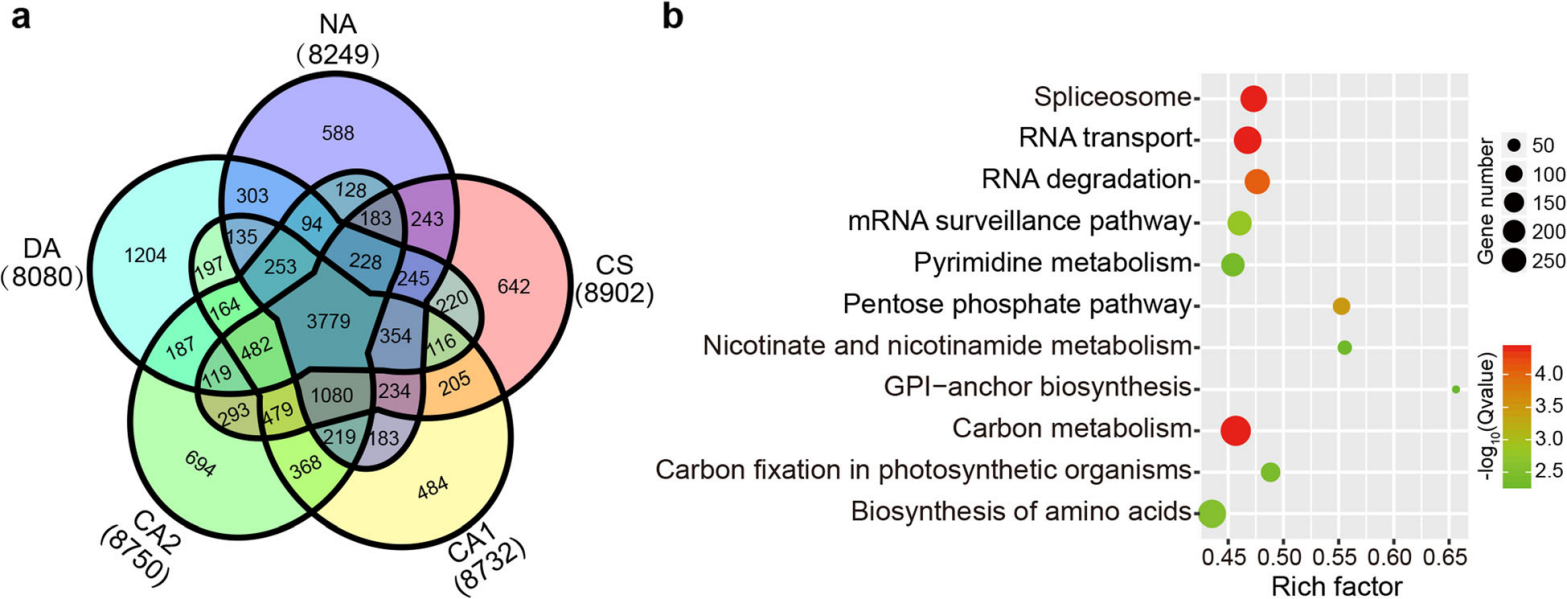
Analysis of AS events at different periods of cold acclimation.**a** Venn diagram showing the common and unique AS genes at different periods of cold acclimation (NA, CS, CA1, CA2, DA). **b** KEGG pathway enrichment for all AS genes (FDR < 0.05). NA: non-acclimation; CS: cold stress for 6 h at 10 °C, day/night; CA1: cold acclimation for 7 days at 10/4 °C, day/night; CA2: cold acclimation for 7 days at 4/0 °C, day/night; DA: de-acclimation for 7 days at 25/20 °C, day/night

Among 14,103 identified AS genes at five different time points, 3779 AS genes were conserved during cold acclimation (Fig. 3a). Some genes were also found to exhibit AS specificity; for example, 1204 DA-specific AS genes were found in DA (de-acclimation). Interestingly, the amount of DA-specific AS genes were significantly more than the number of NA- (588), CS- (642), CA1- (484) and CA2-specific genes (694), suggesting the stimulation of abundant novel AS genes by cold acclimation. To explore the biological functions influenced by AS, KEGG enrichment analyses of 14,103 AS genes were performed (Fig. 3b). The result indicated that the process of spliceosome, RNA transport and RNA degradation were highly enriched, which were in accordance with a previous study [29].

### Dynamic characterization and analysis of AS events during cold acclimation

**Fig. 4:**
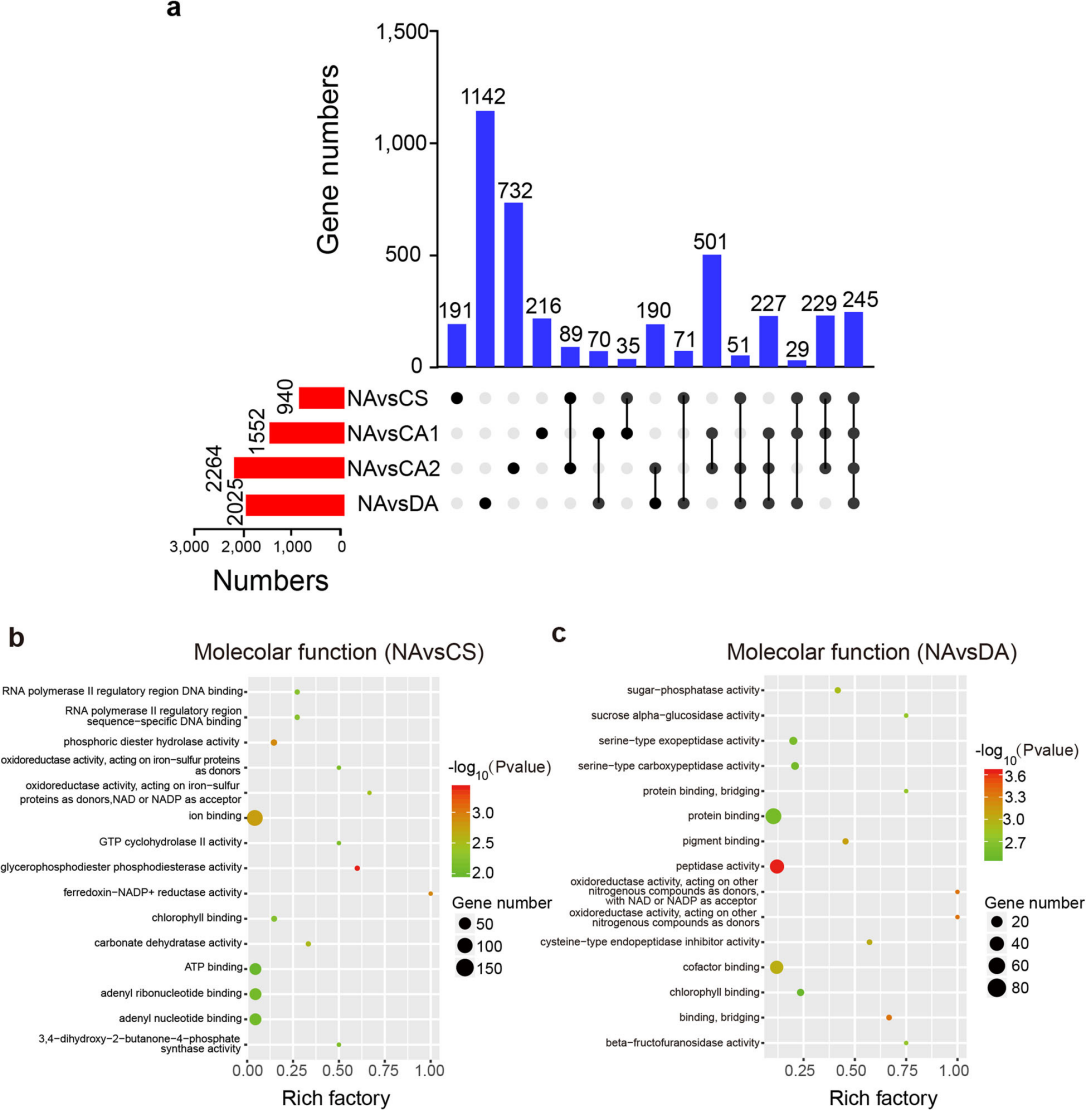
Variation and functional analysis of differentially AS genes (DAGs). **a** Numbers of DAGs and overlap of AS genes among all time points compared with non-acclimation. **b** Gene Ontology (GO) enrichment of DAGs of NA vs CS and NA vs DA in cold acclimation (*p* < 0.01). NA: non-acclimation; CS: cold stress of 6 h at 10 °C, day/night; CA1: cold acclimation of 7 days at 10/4 °C, day/night; CA2: cold acclimation of 7 days at 4/0 °C, day/night; DA: de-acclimation of 7 days at 25/20 °C, day/night

To investigate variation in types of AS events during cold acclimation, we calculated the proportion of the four main AS events (IR, A5SS, A3SS and ES) among the five periods. However, the differences observed among these AS events were insignificant (Additional file 10: Figure S2). We then counted the number of differentially AS genes (DAGs) at different time points (Fig. 4) based on TPM value. It was demonstrated that the number of DAGs gradually increased during cold acclimation (Fig. 4a), with a total of 940, 1552, 2264 and 2025 DAGs observed in the NA vs CS, NA vs CA1, NA vs CA2 and NA vs DA groups, respectively. Notably, the number of unique DAGs (1142) in NA vs DA group was far greater than in other groups, which implied the occurrences of novel specific AS genes in response to DA.

To further determine the biological functions of tea plant AS genes involved in the two transition periods (normal temperature to low temperature and de-acclimation to non-acclimation), we performed GO enrichment analyses of DAGs in these two time points. The highest enrichment of 15 molecular functions is shown according to the *p*-value (*p* < 0.01) (Fig. 4b and c). When the tea plants were initially subjected to cold stress, the DAGs were mainly enriched on the activity of oxidoreductase and the binding of substances. Interestingly, pathways relating to sugar, serine and oxidoreductase activity were significantly enriched after cold acclimation compared with non-acclimation.

### Characterization and expression pattern of AS genes during cold acclimation

To better understand the putative impact of AS during cold acclimation, AS genes associated with cold stress were investigated. We explored and identified genes involved in regulatory and metabolic pathways, including the ICE-CBF-COR pathway, proline metabolism, sugar metabolism pathway, oxidoreductase, abscisic acid pathway these are all involved in the defense response to cold stress. Expression levels of many transcription factors including *CsMYBs*, *CsbHLHs* and *CsWRKYs* were changed at low temperature. (Additional file 2: Table S2). Overall, about 35.9% (42/117) AS events were dramatically enriched in genes within the sugar metabolism pathway, while only 18.6% (24/129) AS events were observed in oxidoreductase clusters (Additional file 2: Table S2). The expression patterns of these AS transcripts were then analyzed (Additional file 11: Figure S3).

**Fig. 5:**
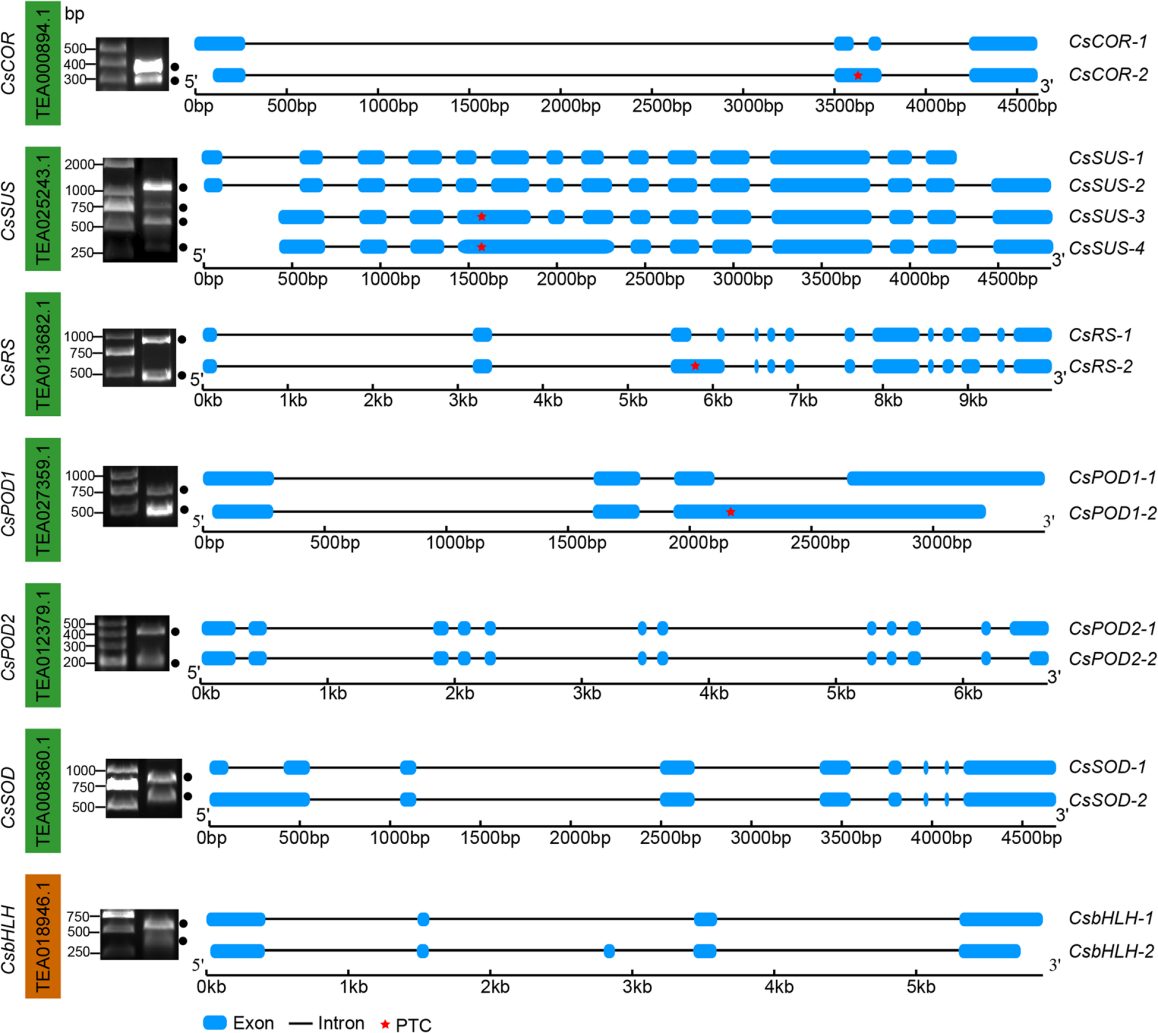
Alternatively spliced isoforms associated with cold stress in tea plant. Red asterisks indicate the position of PTCs. AS transcripts on gel images are denoted with black circles. *CsCOR*: cold regulated gene; *CsSUS*: sucrose synthase gene; *CsRS*: raffinose synthase gene; *CsPOD*: peroxidase gene; *CsSOD*: superoxide dismutase gene; *CsbHLH*: basic helix-loop-helix

To verify the accuracy of the high throughput RNA-seq data, some AS transcripts were selected for validation using RT-PCR and with primers flanking the AS site (Fig. 5 Additional file 3: Table S3). All amplified PCR products were sequenced and verified (Additional file 3 Table S3) which were in consistent with the results of gels. These AS events mainly presented as IR, A3SS and complex types. Among them, IR was the dominant type, and sequence analysis revealed that most IR type AS transcripts could result in introduction of a premature stop codon (PTC); these transcripts may be translated into truncated proteins, thereby imparting structural and functional diversity between AS and non-AS transcripts. For instance, four different AS transcripts were found in *SUS* (sucrose synthase) gene, its full-length transcript (genome annotated transcripts) encodes a protein possessing 838aa and a complete sucrose synthase domain which potentially exhibits catalytic activity. Nevertheless, a PTC was observed in one *SUS* AS transcript (*CsSUS-3*) which encodes only 206 aa of the protein, implying that this remarkable variation may significantly affect the structure and function of this protein (Fig. 5). Additionally, similar truncated proteins were found in *CsCOR* (cold-regulated gene), *CsRS* (raffinose synthase gene) and *CsPOD* (peroxidase gene).

**Fig. 6:**
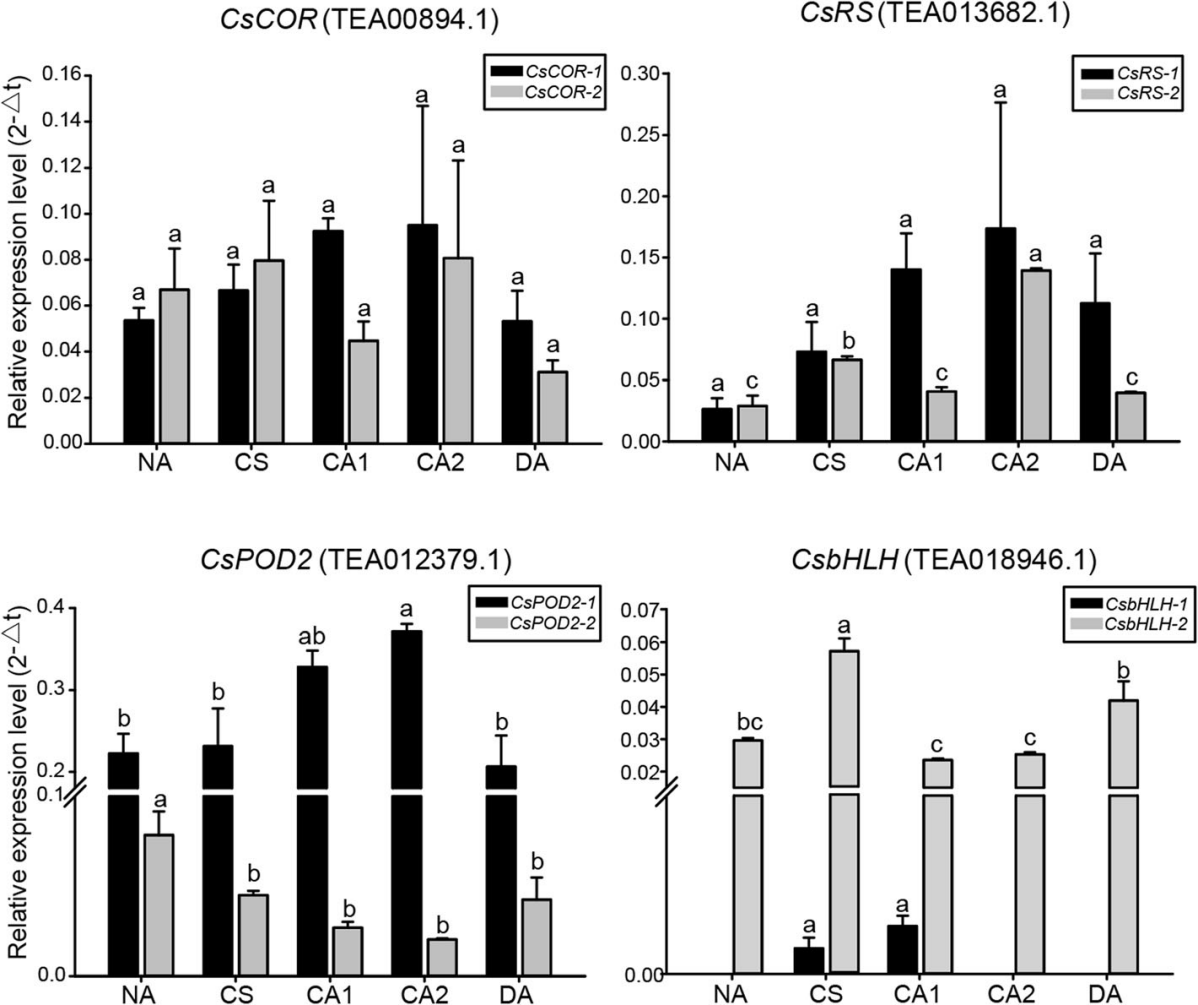
Expression of AS transcripts analyzed by qRT-PCR during acclimation. Black bars and grey bars represent full-length and AS transcripts, respectively. Different letters above the bars represent significant differences at *p* < 0.05. The bars are standard deviations (SD) of two biological replicates. NA: non-acclimation; CS: cold stress of 6 h at 10 °C, day/night; CA1: cold acclimation of 7 days at 10/4 °C, day/night; CA2: cold acclimation of 7 days at 4/0 °C, day/night; DA: de-acclimation of 7 days at 25/20 °C, day/night

To further explore the biological function of these AS transcripts during cold acclimation, their expression patterns were determined using qRT-PCR. The expression levels of AS transcripts vary significantly among different time periods (Fig. 6). For example, the expression of full-length *CsPOD2* increased under low temperature while its AS isoform showed an opposite expression pattern; expression of the *CsRS-2* isoform was significantly higher during CS and CA2 than the other time periods. Meanwhile, *CsbHLH-1* expressed in CS and CA1 but not the other time periods. The AS isoform *CsbHLH-2* was the predominant transcript and expression was higher under low temperature (Fig. 6).

### The relationship between AS transcripts and metabolites during cold acclimation

**Fig. 7:**
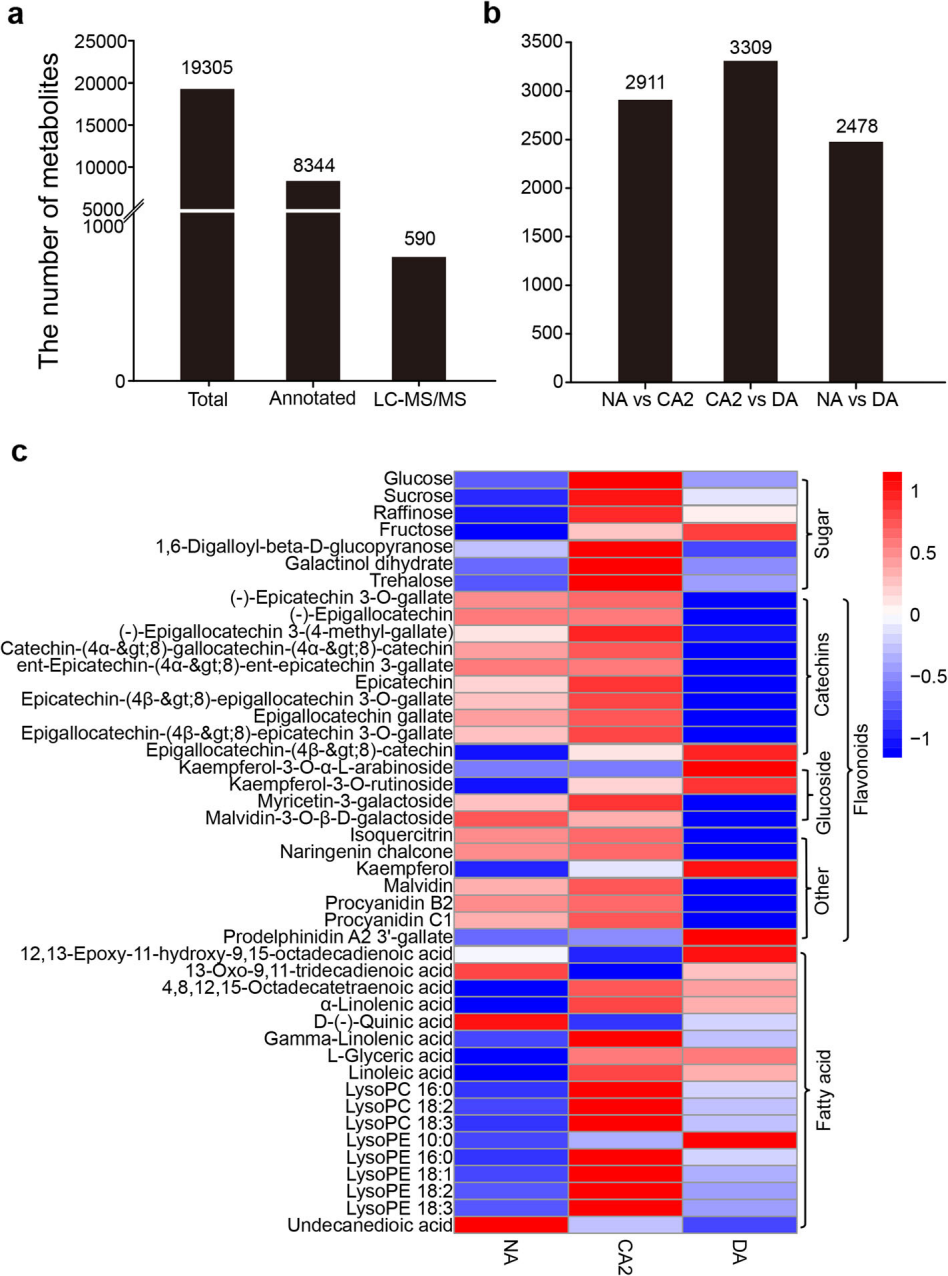
Characteristics of metabolites during cold acclimation. **a** Statistics of total, annotated and LC-MS/MS identified metabolites. **b** Statistics of different metabolites in NA vs CA2, CA2 vs DA and NA vs DA. **c** Relative accumulation of metabolites from six replicates is shown in the heat map generated with R software. Blue and red bars indicate lower and higher expression levels, respectively. The names and types of metabolites are shown on the left and right sides. NA: non-acclimation; CA2: cold acclimation of 7 days at 4/0 °C, day/night; DA: de-acclimation of 7 days at 25/20 °C, day/night

To explore the relationship between AS transcripts and metabolites, the metabolome data was initially obtained by LC–MS using the same treated leaf materials with six independent biological replicates. A total of 19,305 substances were identified, including 8344 annotated metabolites another 590 metabolites which were further identified by LC-MS/MS (Fig. 7a). Furthermore, 5062 metabolites with > 2-fold change and *p*-value < 0.05 were defined as differential metabolites; of these, we identified 2911, 3309 and 2478 differential metabolites in the NA vs CA2, CA2 vs DA and NA vs DA groups, respectively (Fig. 7b). In addition, a total of 137 differential metabolites were finally identified and classified by LC-MS/MS, of which the majorities were flavonoids, sugars and fatty acids. We analyzed the changes of these three metabolite categories together with glucose, fructose, sucrose and raffinose measured in the previous study [28] during cold acclimation (Fig. 7c).

**Fig. 8:**
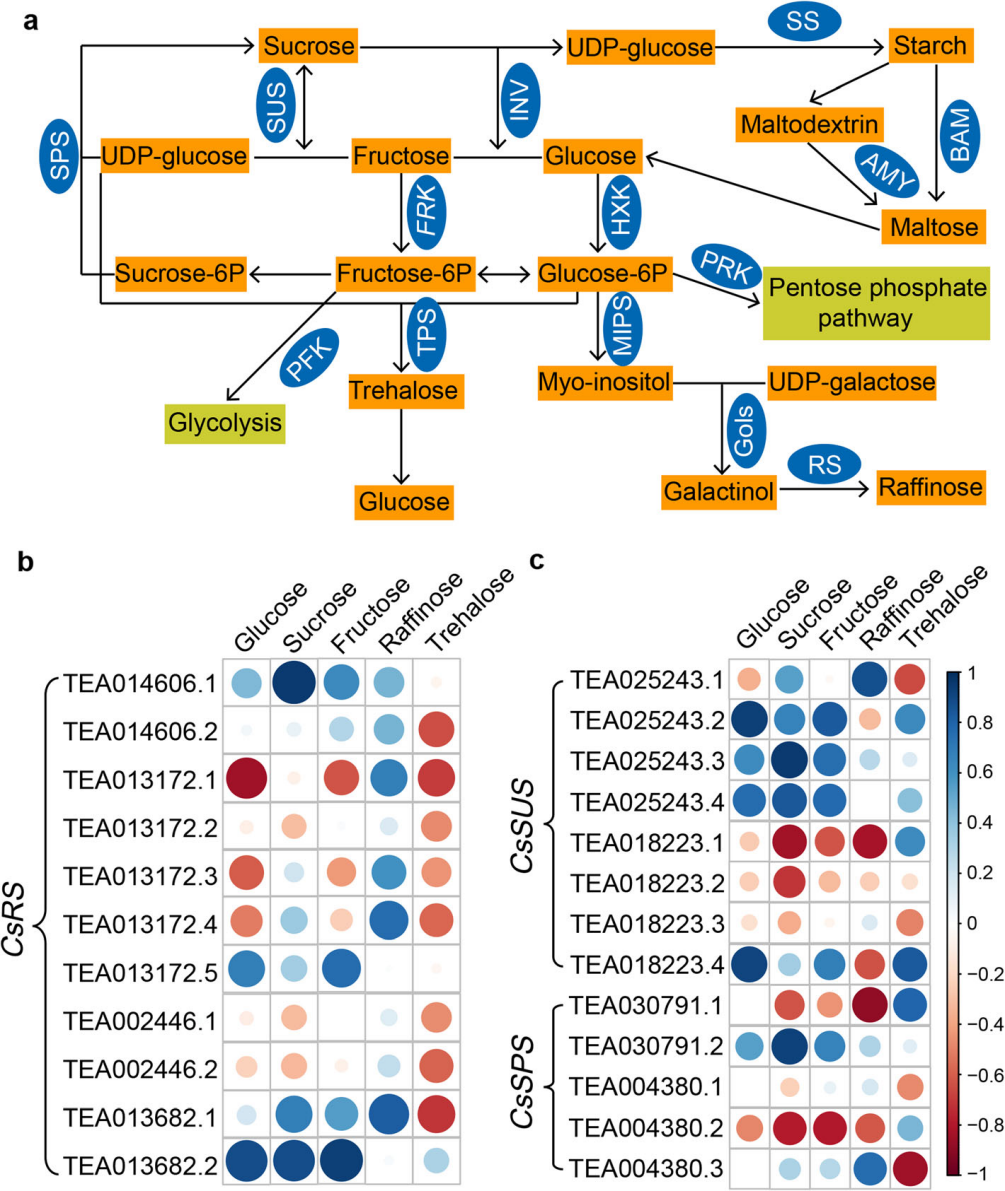
Correlation analysis of AS transcripts and sugar content. **a** The sugar metabolism pathway and AS genes. **b** Transcripts of *CsRS* and sugar content. **c** Transcripts of CsSUS and CsSPS and sugar content. Blue and red circles denote positive and negative correlations, respectively; circle darkness and size indicate correlation strength. SPS: sucrose phosphate synthase; SUS: sucrose synthase; INV: invertase; FRK: fructokinase; HXK: hexokinase; PFK: phosphate fructokinase; TPS: trehalose-6-phosphate synthase; MIPS: myo-inositol phosphate synthase; PRK: phosphoribulokinase; Gols: galactinol synthase; RS: raffinose synthetase; SS: starch synthase; AMY: alpha-amylase; BAM: beta-amylase; *CsRS*: raffinose synthase gene; *CsSUS*: sucrose synthase gene; *CsSPS*: sucrose-phosphate synthase gene

We found that a large number of AS variants were involved in sugar metabolism (Fig. 8a), and sugars were also among the metabolites with differential abundances. Therefore, correlation analysis was performed between these AS transcripts and the contents of sugars. The results showed differences in the correlation between sugar content and AS transcripts (Fig. 8 and Additional file 12: Figure S4). For example, four transcripts of *CsSUS* (TEA025243) were positively correlated with sucrose content; among them, three transcripts (TEA025243.2, TEA025243.3 and TEA025243.4) were positively correlated with glucose and fructose content (Fig. 8c). Moreover, the expression levels of two *SPS* transcripts (TEA030791) showed opposite trends with sucrose content (Fig. 8c). Interestingly, the full-length *CsRS-1* (TEA013682.1) is strongly correlated with raffinose content, while its AS isoform *CsRS-2* (TEA013682.2) is correlated with levels of glucose, sucrose and fructose rather than raffinose (Fig. 8b). We also analyzed the domains of *CsSUS* and *CsRS* transcripts and found that the domains of TEA025243.3, TEA025243.4 and TEA013682.2 were incomplete due to the premature termination codon. What is more interesting is that the TEA025243.2 transcript of *CsSUS* gene possessed a transmembrane region (Additional file 13: Figure S5).

## Discussion

Alternative splicing (AS) plays an important role in plant responses to biotic and abiotic stresses such as cold stress and pathogen defense [13, 33]. In tea plant, previous studies indicate AS is involved in important secondary metabolism pathways such as flavonoid and linalool biosynthesis [29, 34]. Cold stress is one of the most severe abiotic stresses in tea plant, and a series of studies have been focusing on tea plant gene regulation during cold stress and cold acclimation [27, 35, 36]. Through cold acclimation, tea plants can alleviate the harm of low temperature and enhance their tolerance to cold stress [26]. However, little information is known about AS in tea plant during cold acclimation. In this study, we investigated the total AS events during cold acclimation in tea plant through high-throughput RNA-seq. The numbers and expression levels of AS events varied greatly during cold acclimation, and some AS transcripts were strongly correlated with sugar content (correlation coefficient > 0.7). This study provides novel understanding of the roles of AS for cold acclimation in tea plant.

Previous studies have reported that AS changes are influenced by developmental and environmental factors [37, 38]. For instance, during the development of seed, endosperm and embryo, the AS isoforms change significantly in maize [39]. In wheat, 251, 6618 and 7541 AS isoform changes were detected in response to drought stress, heat stress and combined stress in 9312 stress-responsive events, respectively [40]. But little is known about the number of AS changes in response to cold acclimation. During cold acclimation, we found that the number of AS transcripts increased about 3000 in response to low temperature, but decreased by about 6000 during DA (Fig. 2); this implies that low temperature can induce new AS event and AS genes may be adapted to low-temperature environment, which would explain the reduced occurrence of AS events during DA. In addition, we made a comparison with two acclimatedplants of Longjing 43 (SRA database number: SRA061043) and Yinghong 9 (SRA database number: SRP108833), and found that the number of AS was also increased during CA2 in Yinghong 9 and CA3 in Longjing43, but with different extents, probably because the varieties and treatment conditions were different (Additional file 6: Table S6). Low temperature can affect not only the variation of AS number but also the expression level of AS transcripts to generate a large number of DAGs. In our study, the number of DAGs increased gradually with increasing cold acclimation (Fig. 4). This indicates that plants need to produce more functional AS to resist the gradually decreasing temperatures. Similarly in *A.thaliana,* DAGs also change with time at low temperatures, and these DAGs also gradually increased over the course of 4 °C treatment [18]. The variation in the number of DAGs was also reported in drought-treated maize, and the number of DAGs increased significantly after 24 h of drought treatment compared with a 6 h of drought treatment [41]. In addition, the number of DAGs was greater at de-acclimation compared with non-acclimation, which also suggests that the improvement of plant tolerance to low temperature after acclimation may be regulated by a large number of DAGs. To mitigate cold damage, the plant can eliminate reactive oxygen and maintain a redox balance by antioxidant metabolism [9, 42]. Our results showed that the oxidoreductase activity was enriched both at NA vs CS and NA vs DA (Fig. 4). Furthermore, many of the DAGs in NA vs DA are related to sugar metabolism; In *A.thaliana*, sugar metabolism also significantly increased during cold acclimation [5]. Therefore, the ability of tea plant to tolerate low temperatures may be enhanced by changing the number and expression of AS, which thereby regulates oxidoreductase activity and sugar metabolism. Most unique DAGs are obtained from NA vs DA group, while the majority of differences on the metabolic level are in CA2 vs DA group. It is consistent with the reality that change of metabolic levels was lagged in comparison with the change of gene expression during transcription. Moreover, metabolite levels are often affected by various regulators at the same time in a complex regulate network, and the similar inconsistency between metabolites and transcripts has been reported [5].

The occurrence of AS is influenced by low temperature. For example, cold-induction resulted in alternative splicing of *MtJMJC5* (JmjC domain-containing proteins) in *Medicago truncatula*, and the AS of *MtJMJC5* could be reversed after the temperature returns to normal [43]. In *A.thaliana*, plant biological clocks are also regulated by temperature associated AS events [17]. Two intron-retained COR genes were previously found to be induced at low temperatures in durum wheat [20]. In this study, we also found that the *COR* genes underwent AS events. In addition, a large number of AS events were found in the sugar metabolism and antioxidant pathways such as *CsSUS*, *CsRS* and *CsPOD*. All of these pathways have been associated with plant cold resistance. The ICE-CBF-COR regulatory pathway is one of the major ways how plants acquire resistance to cold [44, 45]. Plants can also protect their membrane structures and reduce cold damage through the synergistic action of superoxide dismutase (SOD), peroxidase (POD) protective enzymes and sugar metabolism [46, 47]. Our results suggest that AS may play crucial regulatory roles in these pathways in order to enhance the cold resistance of plants. Moreover, the tobacco transcription factor *NtbHLH123* positively regulates the *CBF* Pathway and reactive oxygen species homeostasis to impart resistance to cold stress [48]. However, the effect of AS on *bHLH* transcription factors during cold acclimation has not been previously explored. Most significantly, the *CsbHLH* isoforms were the predominant transcripts under cold treatments, and had higher expression levels than the full-length transcripts; this implies that the two AS isoforms may play a more important regulatory role and function for cold stress. As a precedent for this hypothesis, the *Athaliana* AS isoform *FLM-β* (*β* isoform of FLOWERING LOCUS M, a MADS-box transcription factor gene) is predominately formed at low temperatures and prevents precocious flowering by interacting with *SVP* (the Short Vegetative Phase gene, an MADS-box transcription factor) [12]. The *bHLH104* transcription factor has been reported to be involved in the sugar and ABA signaling pathways in *A.thaliana* [49]. We found that the *bHLH* gene of TEA018946.1 has a high sequence similarity with that of *A.thaliana*, and thus, it was speculated that this *bHLH* AS isoform may be involved in some signaling pathways and in the downstream gene expression regulation during cold acclimation. The same variation trends of expression levels of this *bHLH* were also found in the acclimation tea plant of longjing43 and yinghong 9 under cold condition (Additional file 7: Table S7). In addition, The comparison of the AS events in the sugar metabolism and antioxidant pathways between Shuchazao and Longjing43 or Yinghong 9 was made respectively, and it was found that many AS events were the same (Additional file 8: Table S8).

It is well established that metabolites contents changed dramatically during cold acclimation [50, 51]. For example, most flavonols and anthocyanins accumulated during cold acclimation in *A.thaliana* [52], and the accumulation of trienoic fatty acid during acclimation is one of the necessary conditions for normal development of leaves at low temperature in tobacco [7]. We found that sugar, flavonoids and fatty acids were three major classes of metabolites, and almost all of them were increased at low temperature, while quinic acid, tridecadienoic acid and octadecadienoic acid decreased. However, these reports only focused on the changes of metabolites and gene expression level during cold acclimation. In comparison, we found that these metabolites may be regulated by AS, and some metabolites remained at higher levels during de-acclimation; therefore, the presence of these metabolites may increase the resistance of plants to low temperature after acclimation. In addition, sugar has been widely studied as a class of metabolites playing a role in cold acclimation [5, 53]. In tea plant, the effects of cold acclimation on sugar metabolism and sugar-related gene expression has been reported [27], and in their study, the expression profiles of a large number of sugar-related genes were verified. However, a large number of AS events in sugar-related genes have not been reported. In our correlation analysis of AS and sugar content, levels of the full-length transcript of *CsRS-1* (TEA013682.1) had a significant positive correlation with raffinose levels, while levels of its AS isoform *CsRS-2* (TEA013682.2) were positively correlated with glucose, sucrose and fructose (Fig. 8). It is possible that the truncated proteins formed by the retention of introns did not have the catalytic activity of raffinose synthase, and the absence of raffinose promoted the synthesis of other soluble sugars. Changes in the functions of truncated proteins have also been reported in *A.thaliana*. For example, the *JAZ* gene can generate truncated protein due to the retention of introns, which weakens the interaction with JA receptor CORONATINE INSENSITIVE1 in the presence of the active JA-Ile conjugate; this imparts resistance to proteasomal degradation [54, 55]. We found that AS transcripts and full-length transcript of *SUS* have different correlations with metabolites and their domain were changed. This suggests that changes in the domain cause changes in gene function. For example, AS transcripts HAB1.1 and HAB1.2 play opposing roles in ABA-mediated seed germination and ABA-mediated post-germination developmental arrest in *A. thaliana* [56].

## Conclusions

**Fig. 9:**
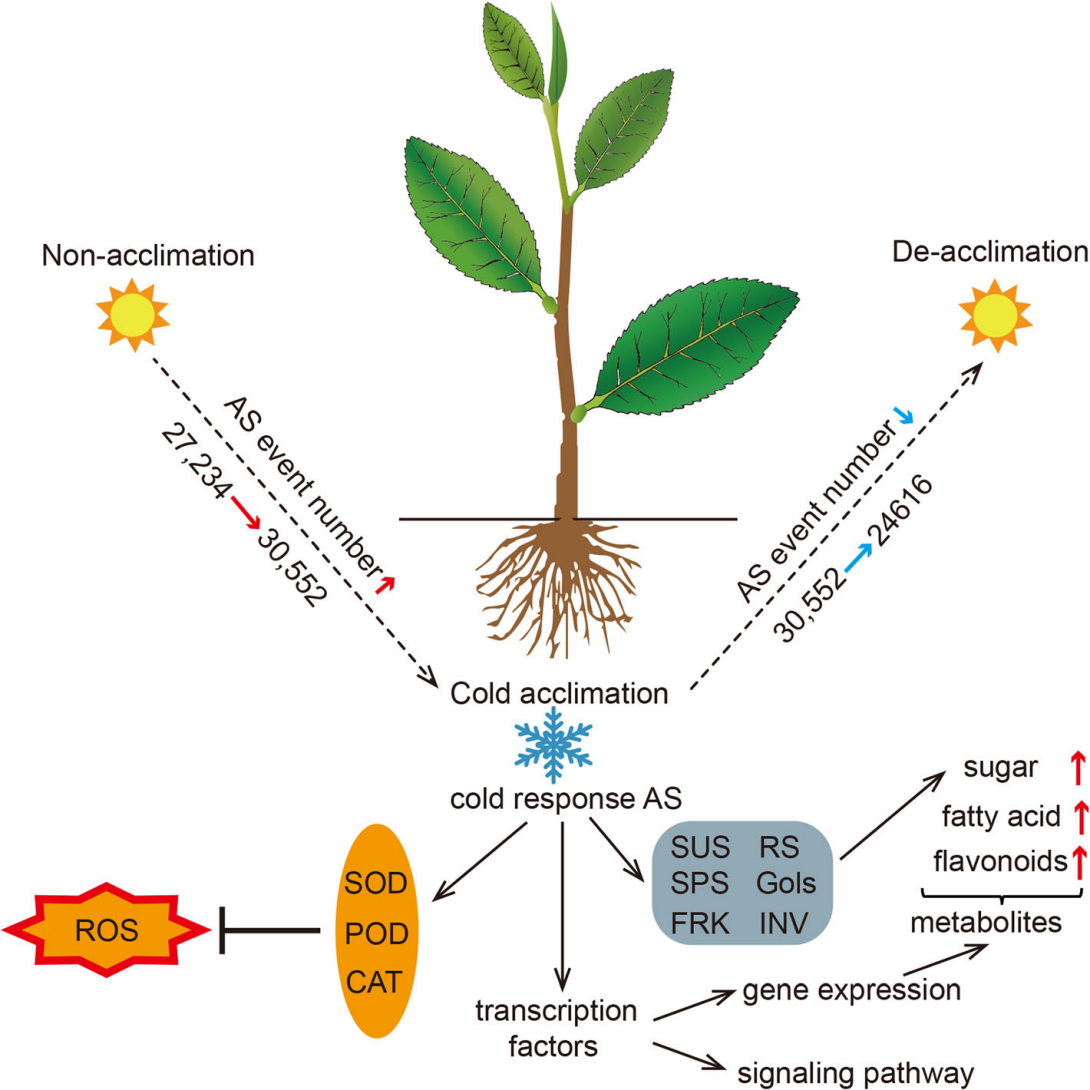
Regulation model of alternative splicing (AS) transcripts in cold acclimation. The red and blue arrows indicate increased and decreased levels, respectively. SOD: superoxide dismutase; POD: peroxidase; CAT: catalase; ROS: reactive oxygen species; SUS: sucrose synthase; RS: raffinose synthase; SPS: sucrose phosphate synthase; Gols: galactinol synthase; FRK: fructokinase; IVN: invertase

In conclusion, we analyzed the AS events during cold acclimation of tea plant based on the transcriptome. The results of this study revealed the dynamic changes in AS and its possible regulatory function during cold acclimation. Based on these results, we propose that changes in abundances and expression levels of AS transcripts may influence sugar metabolism and the antioxidant enzyme system to impart resistance to cold stress. Meanwhile, some transcription factors may regulate downstream genes or signal pathways through AS (Fig. 9). This study provides an essential insight into AS events involved in tea plant cold acclimation, and highlights the importance of AS regulatory function during low temperature stress.

## Methods

### Plant materials and growth conditions

One-year-old clone plants propagated with cuttings of (*Camellia sinensis var. sinensis* ‘Shuchazao’) grown in soil were obtained from the Dechang Tea Plantation in in Shucheng County, Anhui Province (116° 56′ 24″ E, 31° 27′N), China. The tea plant ‘Shuchazao’ (accession no. GS2002008; Agricultural Plant Variety Name Retrieval System, Ministry of Agriculture and Rural Affairs, China) is a national tea cultivar of China, which belongs to the subspecies *Camellia sinensis var. sinensis* of *Camellia sinensis* (L) O. Kuntze. Then the tea plants (30-40 cm) were transferred to a growth chamber at temperatures of 25 °C/20 °C for daytime/night-time. The culture conditions and treatments were described previously [28]. The tea plants were subjected to cold acclimation and de-acclimation with the treatments as follows: Ten well-grown plants were collected for treatments of non-acclimation (NA), CS (sampling after 10 °C for 6 h), CA1 (sampling after 10/4 °C, day/night for 7 days), and CA2 (sampling after 4/0 °C, day/night for another 7 days). Finally, the treated plants of Shuchazao were placed at normal temperature (25/20 °C, day/night temperature) for 7 days of de-acclimation (DA). The leaf samples (CA1, CA2, DA) were collected at the same time (9:00 am). Three biological leaf samples were obtained at each time point. All the samples were immediately frozen in liquid nitrogen and then maintained at − 80 °C. However, the leaf samples were used in this experiment only had two biological replicates due to the consumption during previous experiments.

### RNA extraction and sequencing

Total RNA was extracted from tea leaves using the Total RNA Purification Kit (NorgenBiotek Corporation, Canada) according to the manufacturer’s protocol. The quality and quantity of each RNA extract was determined using agarose gel electrophoresis and the Nanodrop 2500 (Thermo Fisher Scientific, US). RNA-Seq libraries were constructed as described previously [57] and Paired-end 150-bp sequencing was performed for the qualified library on a HiSeq X Ten machine. During sequencing, mRNA was enriched from total RNA using magnetic beads with Oligo (dT). Reads containing low-quality (low quality base, linker contamination and with unknown base N) were filtered by SOAPnuke (version 1.5.6) software [58], and the remaining reads (clean sequencing data) were used for subsequent analyses. The specific parameters are as follows: (1) Removing the reads containing the adapters (−f, −r); (2) Removing reads with unknown base N greater than 5% (−n); (3) Removing low-quality reads (defined as those containing > 20% bases with a quality value < 10) (−l, −q). The RNA-Seq data are publicly available at the SRA database of NCBI (https://www.ncbi.nlm.nih.gov/) under project accession number PRJNA387105.

### Alignment and analysis of the sequencing data

The clean sequencing data were mapped to the tea plant reference genome [59] using HISAT2 (v.2.0.5) with default parameter. Transcripts were assembled by StringTie software (v.1.3.3b) with default settings. The transcripts per million reads (TPM) were calculated by StringTie and applied to quantify the transcript expression level. Ultimately, the assembled transcripts were aligned to the annotation by using Cuffcompare [60], and only transcripts with class_code ‘=’ or ‘j’ were considered as isoforms of known genes.

For the genic splice junctions, we took those coming from protein-coding genes and examined the predicted splice junctions within the coding sequence, 5′UTR or 3′UTR. If one extremity of the junction was in the coding sequence and the other in the UTR, they were classified as 5′UTR-CDS or CDS-3′UTR. The branch site and polypyrimidine tract for each intron were identified using a motif searching method implemented in a self-written PERL script, as described in previous study [61]. Sequence logos were created using WebLogo 3 (http://weblogo.threeplusone.com/) [62, 63].

Differential expression analysis was performed using the DEGseq R package (1.34.1) [64]. The analysis results using q-values < 0.01 and fold-change > 2 were considered to be differentially expressed. We defined these differentially expressed genes that undergo AS as differentially expressed AS genes (DAGs). Gene Ontology (GO) and Kyoto Encyclopedia of Genes and Genomes (KEGG) analysis were conducted using OmicShare tool (http://omicshare.com/). GO terms with corrected *P* < 0.01 were considered significantly enriched by differential expression of AS genes and KEGG analysis with the Fisher’s exact test and false discovery rate (FDR) correction, visualizing the enrichment result with the R (v3.3.2) software. Correlation analysis of sugar levels and AS transcripts was also performed by R with ‘corrplot’ package (0.84).

### Characterization of alternative splicing

To identify the type of AS events, the AStalavista tool [65] (http://astalavista.sammeth.net/) was employed using the gtf files assembled from the sequencing data by StringTie. Major types of AS events including intron retention (IR, AS code: 1^2-,0), exon skipping (ES, AS code: 1–2^, 0), alternative 3′ splice site (A3SS, AS code: 1-,2-), alternative 5′ splice site (A5SS, AS code: 1^,2^), IR1 + IR2 (AS code: 1^2–3^4-,0), A5SS or A3SS (AS code: 1^3-,2^4-), IR1 or IR2 (AS code: 1^2-,3^4-), A5SS + A3SS (AS code: 1^4-,2^3-), ES1 + ES2 (AS code: 1–2^3–4^,0), mutually exclusive exons (MXE, AS code: 1–2^,3–4^), A5SS + ES + A3SS (AS code: 1^6-,2^3–4^5-) were extracted from the output files and counted, respectively.

### Validation of alternative splicing

Total RNA was isolated from tea plants at different time points of cold acclimation as described above. First-strand cDNA was synthesized using a PrimeScript RT Reagent Kit (cat 6110A, Takara, Japan) according to the manufacturer’s instructions. The alternative splicing isoforms associated with cold stress were viewed using IGV software [66]. Gene structures were analyzed with online website Gene Structure Display Server (GSDS 2.0, http://gsds.cbi.pku.edu.cn/index.php). The AS transcripts were validated by RT-PCR according to a previous study [29]. Details of all the relevant primers are listed in Additional file 5: Table S5. Conserved domain search services (CD Search, https://www.ncbi.nlm.nih.gov/Structure/cdd/wrpsb.cgi) and SMART (http://smart.embl-heidelberg.de/) was used to predict the domain of AS transcripts with the protein sequences.

### Quantitative real-time PCR (qRT-PCR)

For qRT-PCR, first-strand cDNA was synthesized from total RNA with the PrimeScript RT Reagent Kit (cat RR036A, Takara, Japan) using the manufacturer’s protocols. PCR amplification and thermal cycling conditions were performed according to our previous study [67]. The *CsGAPDH* gene was selected as the internal control. The relative gene expression values were analyzed using the 2^-△Ct^ method [68]. All reactions were run in technical triplicates for each sample. The relevant primers are listed in Additional file 5: Table S5.

## Supplementary information

**Additional file 1: Table S1.** The number and percentages of splicing donor-acceptor di-nucleotide usages among all transcripts.

**Additional file 2: Table S2.** Characteristics of cold stress-responsive AS genes in tea plant.

**Additional file 3: Table S3.** The genome and sequencing sequence of the AS transcript.

**Additional file 4: Table S4.** The data for each sequencing sample.

**Additional file 5: Table S5.** Sequences of primer pairs used for verifying AS transcripts and qRT-PCR analysis.

**Additional file 6: Table S6.** Alternative splicing analysis of Longjing 43 and Yinghong 9.

**Additional file 7: Table S7.** Expression of bHLH in Shuchazao, Longjing 43 and Yinghong 9.

**Additional file 8: Table S8.** Comparison of AS events in Shuchazao, Longjing43 and Yinghong 9.

**Additional file 9: Figure S1.** The picture of tea plantation and tea plants.

**Additional file 10: Figure S2.** Percentages of four main AS types during cold acclimation.

**Additional file 11: Figure S3.** Expression analysis of AS transcripts during cold acclimation.

**Additional file 12: Figure S4.** Correlation analysis of AS transcriptions and sugar content.

**Additional file 13: Figure S5.** Domain analysis of *SUS* and *RS* in AS transcripts.

**Additional file 14: Figure S6.** Expression analysis of qRT-PCR and sequencing results.

**Additional file 15: Figure S7.** Verification of alternatively spliced isoforms.

## Data Availability

The data sets supporting the results of this article are available at the SRA database of NCBI (https://www.ncbi.nlm.nih.gov/) under project accession number PRJNA387105.

## Abbreviations

3′UTR: 3′ untranslated region
5′UTR: 5′ untranslated region
A3SS: Alternative 3′ splice site
A5SS: Alternative 5′ splice site
AS: Alternative splicing
CA: Cold acclimation
CA1: Cold acclimation of 7 days at 10/4 °C, day/night temperature
CA2: Cold acclimation of 7 days at 4/0 °C, day/night temperature
CBF: C-repeat-binding factor
CDS: Coding sequence
COR: Cold-regulated
CS: Cold stress of 6 h at 10 °C, day/night temperature
DA: De-acclimation of 7 days at 25/20 °C, day/night temperature
DAGs: Differentially AS genes
DHN: Dehydrin
ES: Exon skipping
GO: Gene Ontology
IR: Intron retention
KEGG: Kyoto Encyclopedia of Genes and Genomes
LOX: Lipoxygenase
NA: Non-acclimation
NMD: Nonsense-mediated decay
P: Phosphorus
PEPi: Peptide interference
POD: Peroxidase
PTC: Premature stop codon
qRT-PCR: Quantitative real time polymerase chain reaction
RS: Raffinose synthase
RT-PCR: Reverse transcription polymerase chain reaction
SJs: Splicing junctions
SR: Serine/arginine-rich
SUS: Sucrose synthase
