# Supplementary material for: Comprehensive profiling of alternative splicing landscape during cold acclimation in tea plant
Source: BMC Genomics. 2020 Jan 20;21:65. doi: 10.1186/s12864-020-6491-6 (PMC6971990; doi:10.1186/s12864-020-6491-6)
Supplement: Supplementary file 3 — Additional file 3: Table S3. The genome and sequencing sequence of the AS transcript. [file 12864_2020_6491_MOESM3_ESM.docx]

Table S3. The genome and sequencing sequence of the AS transcript.

Genome sequence:

>COR-1

CCCCCCCCCTCTCTCTCTCTCTCTCTCTCTCTCTCTCTCTATATATATATATATATGTTGATTTCACATAGAGAACATCGGATCGGAGAGAAAAATGGTTAAGAACAATTATTTGGCAATGAAGACGACGGGTTCAACAACTTCAACGGAGTTGATAAGTTCTGATCTGCAAGATCTGGGGAACGCCGCAAAGAAGTTCGCAAGTCATGCCATCATGCTCACTTCTGGCCTCGGTCTGGGCTCTGTTCTTCTTCAATGGATAGCTTCAATCGCTGCTATTTATTTGTTGGTTTTGGATCGAACAAACTGGAGGACCAACATTCTTACCGCACTTTTAATCCCATACATTTTCTTCAGTCTTCCTTCATTAGTGTTCAGCTTGCTCAGTGGAGAGATTGGAAAATGGATTGCTTTCATCGCTGTGGTATTGCGTCTCTTCTTCCCCAAACATTTTCCTGATTGGCTAGAAATGCCAGGAGCATTGATTCTCCTCATCGTGGTAGCTCCAAGCTTGATTGCTGATACACTGAGGGACAGTTTTATCGGTGCTGTAATATGTCTTGCCATCGCATGTTACTTGCTTCAAGAACACATCCGAGCGTCAGGTGGGTTCAGAAATTCCTTCACGAAGAGTAGTGGTGTCTCCAACTCCGTTGGCATAATCATTCTTCTAGTCTATCCTGTCTGGGCTTTGTTTAATGACTTTCTATAGGCACCCCTCTCTCGCAGCTTTGTTGTGTGCATTTCTAAAACAAAATTTTTCTTTTTTTTTTTTTTGGTGTGTGTGTGTGTGTGTTTGATTGGTCAATTTGTTGGCAAAAAACTGTCTGGAC

>COR-2

AAGAACAATTATTTGGCAATGAAGACGACGGGTTCAACAACTTCAACGGAGTTGATAAGTTCTGATCTGCAAGATCTGGGGAACGCCGCAAAGAAGTTCGCAAGTCATGCCATCATGCTCACTTCTGGCCTCGGTCTGGGCTCTGTTCTTCTTCAATGGATAGCTTCAATCGCTGCTATTTATTTGTTGGTTTTGGATCGAACAAACTGGAGGACCAACATTCTTACCGCACTTTTAATCCCATACATTTTCTTCAGTCTTCCTTCATTAGTGTTCAGCTTGCTCAGGTGAAAACGGATTATCTTTGCTACTTTGGCTTCATACTTTAATTTCTTTTTTGCTGCTTTTAATCTGTCTGACAATGCAGTGGAGAGATTGGAAAATGGATTGCTTTCATCGCTGTGGTATTGCGTCTCTTCTTCCCCAAACATTTTCCTGATTGGCTAGAAATGCCAGGAGCATTGATTCTCCTCATCGTGGTAGCTCCAAGCTTGATTGCTGATACACTGAGGGACAGTTTTATCGGTGCTGTAATATGTCTTGCCATCGCATGTTACTTGCTTCAAGAACACATCCGAGCGTCAGGTGGGTTCAGAAATTCCTTCACGAAGAGTAGTGGTGTCTCCAACTCCGTTGGCATAATCATTCTTCTAGTCTATCCTGTCTGGGCTTTGTTTAATGACTTTCTATAGGCACCCCTCTCTCGCAGCTTTGTTGTGTGCATTTCTAAAACAAAATTTTTCTTTTTTTTTTTTTTGGTGTGTGTGTGTGTGTGTTTGATTGGTCAATTTGTTGGCAAAAAACTGTCTGGAC

>SUS-1

CAAAATCATTCGCACTTTCGAAATCTAACATTAGAATGGCTTCAAATATTGCAGTAGTTGACAGCTTGTCCGACGCTCTGAGACAGAGCCGTTACTACATGAAGAGATGCTTTTCTGGGTTTGTAGGAATGGGGAGAAGGTTGATGAAACCCCACCAAATAATGGAAGTAATAGACAAAGCAATCGAGGACAAGCGTGAAAGAGCCAGAGTTTTGGAGGGTTTACTCGGCCAAGTCTTCAGTTCTACCCAGGAGGTGGCTATAAATCCACCTTACGTAGCTTTGGCAGTCAGACAGAGCCCTGGTTTTTGGGAATTTTTTAAGGTGAACGCCAATAGTCTAGAAGTGGATTCGATAACAGCCAAAGACTACTTGAAGCTCAAAGAAATAGTGTACGATGAAAATTGGGCAATGGATAAAAATGCACTGGAAATAGATTTTGGAGCATGTGACTTCTCTACTCCTCGCCTAACCCTTTCTTCTTCTATTGGAAATGGAGTTGACTTCATGTCAAAGTTCATGACCTCAAGGATTAGTGGTGATCTTGAACGTGCAAAGCCTTTGCTTGAGTACTTACTTGCCCTAGATCATCACGGAGAGAATCTTATGATCAATGAGAATATCAACACAGTTTCCAAGCTTCAGGCAGGCTTGATCGTGGCTGATGTTTACGTTTCTGCCCTCCCAAAAAACACTCCTTACCAAAATTTTGAGCAGAAGCTTAAAGAGTGGGGGTTCGAGAAAGGGTGGGGAGAAAACGCAGACAGAGTTAAAGAGACAATGAAGATCCTTTCAGAGATACTACAAGCACCGGACCCAATTAAAATGGAATCGTTCTTCAGAAGGCTTCCAAATATATTCAAAATCGTGATCTTCTCAGTCCATGGCTACTTTGGCCAGTCTGATGTCCTTGGCTTGCCTGATACTGGTGGCCAGGTTGTTTACATTCTAGACCAGGTGAAAGCTTTAGAGGAGGAATTGCTGCTGAGAATTAAGCAGCAAGGACTGAGTGTGAAGCCTCAGATTCTTGTGGTGACTCGTCTCATACCAGATGCAAAAGGGACTAAGTGCAGCGAGGAGATTGAGCCTGTCCTCAACACGACACACTCCCACATCCTTAGAGTCCCATTCAAGACAGACAAAGGGGTTCTGCACCAATGGGATGCTACTTCTAAGGTCCTCAAGCACTTGGAATGCAAACCAGACCTTATACTTGGGAACTACACTGATGGAAACTTGGTGGCATCTCTAATGGCTAGCAAACTTGGAGTCACTCTGGGAACCATTGCTCATGCTTTGGAGAAGACTAAGTATGAAGATTCTGATGTCAAATGGAAAGAGTTGGATCCAAAGTACCACTTCTCGTGCCAATTCACAGCAGACTTGATTGCAATGAATTCAGCCGATTTCATCATTACCAGCACATATCAAGAAATTGCAGGAAGCAAGAAGAGGGCTGGACAATATGAAAGCCACCAGGCATTTACAATGCCAGGCCTTTGCAGAGTAGTTTCAGGCATCAACGTCTTTGATCCAAAGTTCAACATTGCTGCCCCAGGGGCTGAACAAGCAGTCTACTTTCCCTTCACAGAGAAAAACAAGCGATTCACATCATTTCATCCTGCCATTGAAGAACTACTCTATGCTAACAAGGATAACCACGAACACATAGGATTTCTGGCAGACAAGAAGAAACCAATCATCTTTTCGATGGCAAGGCTTGACACGGTGAAGAACATTACTGGATTGGTTGAGTGGTATGGGAAGAACAAAAGGCTTAGGAACTTGGCGAATCTTGTCGTTGTTGCAGGGTTTTTTGATCCATCAAAATCGAAAGATAGGGAAGAAATTGCAGAAATAAATAAAATGCATACTTTGATTGAAAAATACCAACTCAAGGGTCAGATCAGATGGATAGCAGCTCAGACTGATAGGTACCGCAATGGAGAGCTGTATCGGTGTATTGCTGATACAAAGGGGGCTTTTGTGCAGCCTGCATTTTATGAGGCTTTTGGTCTGACAGTTATTGAAGCTATGAACTGTGGATTACCAACTTTTGCGACCAATCAAGGAGGACCGGCAGAGATTATTGTTGATGGGGTCTCGGGTTTCCATGTCGATCCTAACAACGATGATGAATCGAGCAACAAGATTGCTGATTTCTTTGAGAAGTGTAAGGTGGATGCTGAGTATTGGAACAAGGTGTCTCAAGAGGGTCTCAAGCGCATATATGAATGCTACTCGTGGAAGATCTATGCAAATAAGGTCTTGAACATGGGATCTTTATACGGGTTTTGGAGGCAATTGAATAAAGAACAGAAACAAGCTAAGCAAAGATATATTCAACTGTTTTACAATCTCCTATTCAGGAATCGAGCGAAGAGCATAGCCATCCCAAGTGCTGAAGCCCAAAAAACAGCACCAACTCCAACGACCAAACCCCAAGAAACAGCGATGCCAAAACCAAAAGAAACAGAAATACTAAAAGCACAAGAAACAGCAAAAACAGCAATTACTAAACCACCAGAACCTACGGCCAGGCATGTTTCCTAA

>SUS-2

CACTTTCGAAATCTAACATTAGAATGGCTTCAAATATTGCAGTAGTTGACAGCTTGTCCGACGCTCTGAGACAGAGCCGTTACTACATGAAGAGATGCTTTTCTGGGTTTGTAGGAATGGGGAGAAGGTTGATGAAACCCCACCAAATAATGGAAGTAATAGACAAAGCAATCGAGGACAAGCGTGAAAGAGCCAGAGTTTTGGAGGGTTTACTCGGCCAAGTCTTCAGTTCTACCCAGGAGGTGGCTATAAATCCACCTTACGTAGCTTTGGCAGTCAGACAGAGCCCTGGTTTTTGGGAATTTTTTAAGGTGAACGCCAATAGTCTAGAAGTGGATTCGATAACAGCCAAAGACTACTTGAAGCTCAAAGAAATAGTGTACGATGAAAATTGGGCAATGGATAAAAATGCACTGGAAATAGATTTTGGAGCATGTGACTTCTCTACTCCTCGCCTAACCCTTTCTTCTTCTATTGGAAATGGAGTTGACTTCATGTCAAAGTTCATGACCTCAAGGATTAGTGGTGATCTTGAACGTGCAAAGCCTTTGCTTGAGTACTTACTTGCCCTAGATCATCACGGAGAGAATCTTATGATCAATGAGAATATCAACACAGTTTCCAAGCTTCAGGCAGGCTTGATCGTGGCTGATGTTTACGTTTCTGCCCTCCCAAAAAACACTCCTTACCAAAATTTTGAGCAGAAGCTTAAAGAGTGGGGGTTCGAGAAAGGGTGGGGAGAAAACGCAGACAGAGTTAAAGAGACAATGAAGATCCTTTCAGAGATACTACAAGCACCGGACCCAATTAAAATGGAATCGTTCTTCAGAAGGCTTCCAAATATATTCAAAATCGTGATCTTCTCAGTCCATGGCTACTTTGGCCAGTCTGATGTCCTTGGCTTGCCTGATACTGGTGGCCAGGTTGTTTACATTCTAGACCAGGTGAAAGCTTTAGAGGAGGAATTGCTGCTGAGAATTAAGCAGCAAGGACTGAGTGTGAAGCCTCAGATTCTTGTGGTGACTCGTCTCATACCAGATGCAAAAGGGACTAAGTGCAGCGAGGAGATTGAGCCTGTCCTCAACACGACACACTCCCACATCCTTAGAGTCCCATTCAAGACAGACAAAGGGGTTCTGCACCAATGGGTATCCCGGTTTGATGACTACCCTTACCTTGAGAGATTTACCCAGGATGCTACTTCTAAGGTCCTCAAGCACTTGGAATGCAAACCAGACCTTATACTTGGGAACTACACTGATGGAAACTTGGTGGCATCTCTAATGGCTAGCAAACTTGGAGTCACTCTGGGAACCATTGCTCATGCTTTGGAGAAGACTAAGTATGAAGATTCTGATGTCAAATGGAAAGAGTTGGATCCAAAGTACCACTTCTCGTGCCAATTCACAGCAGACTTGATTGCAATGAATTCAGCCGATTTCATCATTACCAGCACATATCAAGAAATTGCAGGAAGCAAGAAGAGGGCTGGACAATATGAAAGCCACCAGGCATTTACAATGCCAGGCCTTTGCAGAGTAGTTTCAGGCATCAACGTCTTTGATCCAAAGTTCAACATTGCTGCCCCAGGGGCTGAACAAGCAGTCTACTTTCCCTTCACAGAGAAAAACAAGCGATTCACATCATTTCATCCTGCCATTGAAGAACTACTCTATGCTAACAAGGATAACCACGAACACATAGGATTTCTGGCAGACAAGAAGAAACCAATCATCTTTTCGATGGCAAGGCTTGACACGGTGAAGAACATTACTGGATTGGTTGAGTGGTATGGGAAGAACAAAAGGCTTAGGAACTTGGCGAATCTTGTCGTTGTTGCAGGGTTTTTTGATCCATCAAAATCGAAAGATAGGGAAGAAATTGCAGAAATAAATAAAATGCATACTTTGATTGAAAAATACCAACTCAAGGGTCAGATCAGATGGATAGCAGCTCAGACTGATAGGTACCGCAATGGAGAGCTGTATCGGTGTATTGCTGATACAAAGGGGGCTTTTGTGCAGCCTGCATTTTATGAGGCTTTTGGTCTGACAGTTATTGAAGCTATGAACTGTGGATTACCAACTTTTGCGACCAATCAAGGAGGACCGGCAGAGATTATTGTTGATGGGGTCTCGGGTTTCCATGTCGATCCTAACAACGATGATGAATCGAGCAACAAGATTGCTGATTTCTTTGAGAAGTGTAAGGTGGATGCTGAGTATTGGAACAAGGTGTCTCAAGAGGGTCTCAAGCGCATATATGAATGCTACTCGTGGAAGATCTATGCAAATAAGGTCTTGAACATGGGATCTTTATACGGGTTTTGGAGGCAATTGAATAAAGAACAGAAACAAGCTAAGCAAAGATATATTCAACTGTTTTACAATCTCCTATTCAGGAATCGAGCGAAGAGCATAGCCATCCCAAGTGCTGAAGCCCAAAAAACAGCACCAACTCCAACGACCAAACCCCAAGAAACAGCGATGCCAAAACCAAAAGAAACAGAAATACTAAAAGCACAAGAAACAGCAAAAACAGCAATTACTAAACCACCAGAACCTACGGCCAGGGAAACTGAGGAGCAGCAGCTTGCCTCCCCAAAAACTGATCGCGCAGGTTGCCCACGCTGTTCATGGGCCATCTTTTGCATCTCTGTCTTTATCATTCTTTACACTTATATAAAGTATTATGGGTTATTCAAAGGACCATGGTCAAATGAGTGATTCTTCAGGAAGAAAAGTTAATAGAAACCTCAAATGTGTAAGCTCAATTCATCAACAAAATTTCCTTGCAGTCTTTATTTGACAGCTATTATATAGATGTTTCATGCTAAAACTTTAAGAGCAGTACTTCATTTGAAGCATGCATGCCTGAAAGGGTGTGGGAAGTTTTTGTGGATGCAATTTACA

>SUS-3

TACACTCATTTGCGAATCAAACATGACCTCATTGACCTAAACTTTGCCACGCAAACCATGCGAAGTTCACTAATTATTTAGCTTGGCTAACAAAATAGGAATTTTTGTTCTATGTCTGGAAAATCAAGGTTTGTAGGAATGGGGAGAAGGTTGATGAAACCCCACCAAATAATGGAAGTAATAGACAAAGCAATCGAGGACAAGCGTGAAAGAGCCAGAGTTTTGGAGGGTTTACTCGGCCAAGTCTTCAGTTCTACCCAGGAGGTGGCTATAAATCCACCTTACGTAGCTTTGGCAGTCAGACAGAGCCCTGGTTTTTGGGAATTTTTTAAGGTGAACGCCAATAGTCTAGAAGTGGATTCGATAACAGCCAAAGACTACTTGAAGCTCAAAGAAATAGTGTACGATGAAAATTGGGCAATGGATAAAAATGCACTGGAAATAGATTTTGGAGCATGTGACTTCTCTACTCCTCGCCTAACCCTTTCTTCTTCTATTGGAAATGGAGTTGACTTCATGTCAAAGTTCATGACCTCAAGGATTAGTGGTGATCTTGAACGTGCAAAGCCTTTGCTTGAGTACTTACTTGCCCTAGATCATCACGGAGAGAATCTTATGATCAATGAGAATATCAACACAGTTTCCAAGCTTCAGGCAGGCTTGATCGTGGCTGATGTTTACGTTTCTGCCCTCCCAAAAAACACTCCTTACCAAAATTTTGAGCAGAAGTAACCATTTCTTCTATGTTCACTGTAAATTATAGTTCTTGTGATCTCATATATTGTGCAAACCTATTTTTGTATTTTCAGGCTTAAAGAGTGGGGGTTCGAGAAAGGGTGGGGAGAAAACGCAGACAGAGTTAAAGAGACAATGAAGATCCTTTCAGAGATACTACAAGCACCGGACCCAATTAAAATGGAATCGTTCTTCAGAAGGCTTCCAAATATATTCAAAATCGTGATCTTCTCAGTCCATGGCTACTTTGGCCAGTCTGATGTCCTTGGCTTGCCTGATACTGGTGGCCAGGTTGTTTACATTCTAGACCAGGTGAAAGCTTTAGAGGAGGAATTGCTGCTGAGAATTAAGCAGCAAGGACTGAGTGTGAAGCCTCAGATTCTTGTGGTGACTCGTCTCATACCAGATGCAAAAGGGACTAAGTGCAGCGAGGAGATTGAGCCTGTCCTCAACACGACACACTCCCACATCCTTAGAGTCCCATTCAAGACAGACAAAGGGGTTCTGCACCAATGGGTATCCCGGTTTGATGACTACCCTTACCTTGAGAGATTTACCCAGGATGCTACTTCTAAGGTCCTCAAGCACTTGGAATGCAAACCAGACCTTATACTTGGGAACTACACTGATGGAAACTTGGTGGCATCTCTAATGGCTAGCAAACTTGGAGTCACTCTGGGAACCATTGCTCATGCTTTGGAGAAGACTAAGTATGAAGATTCTGATGTCAAATGGAAAGAGTTGGATCCAAAGTACCACTTCTCGTGCCAATTCACAGCAGACTTGATTGCAATGAATTCAGCCGATTTCATCATTACCAGCACATATCAAGAAATTGCAGGAAGCAAGAAGAGGGCTGGACAATATGAAAGCCACCAGGCATTTACAATGCCAGGCCTTTGCAGAGTAGTTTCAGGCATCAACGTCTTTGATCCAAAGTTCAACATTGCTGCCCCAGGGGCTGAACAAGCAGTCTACTTTCCCTTCACAGAGAAAAACAAGCGATTCACATCATTTCATCCTGCCATTGAAGAACTACTCTATGCTAACAAGGATAACCACGAACACATAGGATTTCTGGCAGACAAGAAGAAACCAATCATCTTTTCGATGGCAAGGCTTGACACGGTGAAGAACATTACTGGATTGGTTGAGTGGTATGGGAAGAACAAAAGGCTTAGGAACTTGGCGAATCTTGTCGTTGTTGCAGGGTTTTTTGATCCATCAAAATCGAAAGATAGGGAAGAAATTGCAGAAATAAATAAAATGCATACTTTGATTGAAAAATACCAACTCAAGGGTCAGATCAGATGGATAGCAGCTCAGACTGATAGGTACCGCAATGGAGAGCTGTATCGGTGTATTGCTGATACAAAGGGGGCTTTTGTGCAGCCTGCATTTTATGAGGCTTTTGGTCTGACAGTTATTGAAGCTATGAACTGTGGATTACCAACTTTTGCGACCAATCAAGGAGGACCGGCAGAGATTATTGTTGATGGGGTCTCGGGTTTCCATGTCGATCCTAACAACGATGATGAATCGAGCAACAAGATTGCTGATTTCTTTGAGAAGTGTAAGGTGGATGCTGAGTATTGGAACAAGGTGTCTCAAGAGGGTCTCAAGCGCATATATGAATGCTACTCGTGGAAGATCTATGCAAATAAGGTCTTGAACATGGGATCTTTATACGGGTTTTGGAGGCAATTGAATAAAGAACAGAAACAAGCTAAGCAAAGATATATTCAACTGTTTTACAATCTCCTATTCAGGAATCGAGCGAAGAGCATAGCCATCCCAAGTGCTGAAGCCCAAAAAACAGCACCAACTCCAACGACCAAACCCCAAGAAACAGCGATGCCAAAACCAAAAGAAACAGAAATACTAAAAGCACAAGAAACAGCAAAAACAGCAATTACTAAACCACCAGAACCTACGGCCAGGGAAACTGAGGAGCAGCAGCTTGCCTCCCCAAAAACTGATCGCGCAGGTTGCCCACGCTGTTCATGGGCCATCTTTTGCATCTCTGTCTTTATCATTCTTTACACTTATATAAAGTATTATGGGTTATTCAAAGGACCATGGTCAAATGAGTGATTCTTCAGGAAGAAAAGTTAATAGAAACCTCAAATGTGTAAGCTCAATTCATCAACAAAATTTCCTTGCAGTCTTTATTTGACAGCTATTATATAGATGTTTCATGCTAAAACTTTAAGAGCAGTACTTCATTTGAAGCATGCATGCCTGAAAGGGTGTGGGAAGTTTTTGTGGATGCAATTTACA

>SUS-4

TACACTCATTTGCGAATCAAACATGACCTCATTGACCTAAACTTTGCCACGCAAACCATGCGAAGTTCACTAATTATTTAGCTTGGCTAACAAAATAGGAATTTTTGTTCTATGTCTGGAAAATCAAGGTTTGTAGGAATGGGGAGAAGGTTGATGAAACCCCACCAAATAATGGAAGTAATAGACAAAGCAATCGAGGACAAGCGTGAAAGAGCCAGAGTTTTGGAGGGTTTACTCGGCCAAGTCTTCAGTTCTACCCAGGAGGTGGCTATAAATCCACCTTACGTAGCTTTGGCAGTCAGACAGAGCCCTGGTTTTTGGGAATTTTTTAAGGTGAACGCCAATAGTCTAGAAGTGGATTCGATAACAGCCAAAGACTACTTGAAGCTCAAAGAAATAGTGTACGATGAAAATTGGGCAATGGATAAAAATGCACTGGAAATAGATTTTGGAGCATGTGACTTCTCTACTCCTCGCCTAACCCTTTCTTCTTCTATTGGAAATGGAGTTGACTTCATGTCAAAGTTCATGACCTCAAGGATTAGTGGTGATCTTGAACGTGCAAAGCCTTTGCTTGAGTACTTACTTGCCCTAGATCATCACGGAGAGAATCTTATGATCAATGAGAATATCAACACAGTTTCCAAGCTTCAGGCAGGCTTGATCGTGGCTGATGTTTACGTTTCTGCCCTCCCAAAAAACACTCCTTACCAAAATTTTGAGCAGAAGTAACCATTTCTTCTATGTTCACTGTAAATTATAGTTCTTGTGATCTCATATATTGTGCAAACCTATTTTTGTATTTTCAGGCTTAAAGAGTGGGGGTTCGAGAAAGGGTGGGGAGAAAACGCAGACAGAGTTAAAGAGACAATGAAGATCCTTTCAGAGATACTACAAGCACCGGACCCAATTAAAATGGAATCGTTCTTCAGAAGGCTTCCAAATATATTCAAAATCGTGATCTTCTCAGTCCATGGCTACTTTGGCCAGTCTGATGTCCTTGGCTTGCCTGATACTGGTGGCCAGGTATATAACACAACCAGTCACATTAGTATGAGCAATAGTACATACATACAAAGCTCGATGTACAACCAGGACTAACAGGCTTTTGAATTTTCAACAGGTTGTTTACATTCTAGACCAGGTGAAAGCTTTAGAGGAGGAATTGCTGCTGAGAATTAAGCAGCAAGGACTGAGTGTGAAGCCTCAGATTCTTGTGGTGAGCGATGCATAAAATTCACCAAGAAATGATTTAATTGACCGAGAGAAGATAGGTTTAAGAGTTTATAAGTCTTTCTTGCTTTGCTTATATGTCCACAGGTGACTCGTCTCATACCAGATGCAAAAGGGACTAAGTGCAGCGAGGAGATTGAGCCTGTCCTCAACACGACACACTCCCACATCCTTAGAGTCCCATTCAAGACAGACAAAGGGGTTCTGCACCAATGGGTATCCCGGTTTGATGACTACCCTTACCTTGAGAGATTTACCCAGGATGCTACTTCTAAGGTCCTCAAGCACTTGGAATGCAAACCAGACCTTATACTTGGGAACTACACTGATGGAAACTTGGTGGCATCTCTAATGGCTAGCAAACTTGGAGTCACTCTGGGAACCATTGCTCATGCTTTGGAGAAGACTAAGTATGAAGATTCTGATGTCAAATGGAAAGAGTTGGATCCAAAGTACCACTTCTCGTGCCAATTCACAGCAGACTTGATTGCAATGAATTCAGCCGATTTCATCATTACCAGCACATATCAAGAAATTGCAGGAAGCAAGAAGAGGGCTGGACAATATGAAAGCCACCAGGCATTTACAATGCCAGGCCTTTGCAGAGTAGTTTCAGGCATCAACGTCTTTGATCCAAAGTTCAACATTGCTGCCCCAGGGGCTGAACAAGCAGTCTACTTTCCCTTCACAGAGAAAAACAAGCGATTCACATCATTTCATCCTGCCATTGAAGAACTACTCTATGCTAACAAGGATAACCACGAACACATAGGATTTCTGGCAGACAAGAAGAAACCAATCATCTTTTCGATGGCAAGGCTTGACACGGTGAAGAACATTACTGGATTGGTTGAGTGGTATGGGAAGAACAAAAGGCTTAGGAACTTGGCGAATCTTGTCGTTGTTGCAGGGTTTTTTGATCCATCAAAATCGAAAGATAGGGAAGAAATTGCAGAAATAAATAAAATGCATACTTTGATTGAAAAATACCAACTCAAGGGTCAGATCAGATGGATAGCAGCTCAGACTGATAGGTACCGCAATGGAGAGCTGTATCGGTGTATTGCTGATACAAAGGGGGCTTTTGTGCAGCCTGCATTTTATGAGGCTTTTGGTCTGACAGTTATTGAAGCTATGAACTGTGGATTACCAACTTTTGCGACCAATCAAGGAGGACCGGCAGAGATTATTGTTGATGGGGTCTCGGGTTTCCATGTCGATCCTAACAACGATGATGAATCGAGCAACAAGATTGCTGATTTCTTTGAGAAGTGTAAGGTGGATGCTGAGTATTGGAACAAGGTGTCTCAAGAGGGTCTCAAGCGCATATATGAATGCTACTCGTGGAAGATCTATGCAAATAAGGTCTTGAACATGGGATCTTTATACGGGTTTTGGAGGCAATTGAATAAAGAACAGAAACAAGCTAAGCAAAGATATATTCAACTGTTTTACAATCTCCTATTCAGGAATCGAGCGAAGAGCATAGCCATCCCAAGTGCTGAAGCCCAAAAAACAGCACCAACTCCAACGACCAAACCCCAAGAAACAGCGATGCCAAAACCAAAAGAAACAGAAATACTAAAAGCACAAGAAACAGCAAAAACAGCAATTACTAAACCACCAGAACCTACGGCCAGGGAAACTGAGGAGCAGCAGCTTGCCTCCCCAAAAACTGATCGCGCAGGTTGCCCACGCTGTTCATGGGCCATCTTTTGCATCTCTGTCTTTATCATTCTTTACACTTATATAAAGTATTATGGGTTATTCAAAGGACCATGGTCAAATGAGTGATTCTTCAGGAAGAAAAGTTAATAGAAACCTCAAATGTGTAAGCTCAATTCATCAACAAAATTTCCTTGCAGTCTTTATTTGACAGCTATTATATAGATGTTTCATGCTAAAACTTTAAGAGCAGTACTTCATTTGAAGCATGCATGCCTGAAAGGGTGTGGGAAGTTTTTGTGGATGCAATTTACA

>CsRS-1

GTTTTGCCATTTGCCTGTGTTTTAAAATTTCTCCTTCGTTGCGAAGTTTGCATTCCTTTGTGGAATGCGCTTCTGCTTTTAGTTTGATTTCTATCTTCTCTCTGTCTCTCTATTCGTTGATCGGAGCTCGGGAAAAGATTTTCCGGCGAGGCGGCTACAGATACAGAGTTTATTGTGTAGCTAGCAAGAAGTAGGGTGCTGAAAGATGACGGTTACTCCTAATATCTCGGTCAGTGATGGAAACCTTGTGGTTCAGGGGAAGACCATACTGAAGGGTGTGCCCGAAAACATTGTTTTGACTCCGGGACCAGGCGTGGGCCTTGTAGCTGGTGCTTTCATTGGTGCCACTGCTGATCACAGCAAAAGCCTCCATGTCTTCCCCGTGGGCGTCTTAGAGGATCTCCGATTCATGTGTTGTTTTCGTTTCAAGCTATGGTGGATGACTCAGAGAATGGGGACATGTGGGAAGGACATTCCTCTTGAGACACAAATCTTGCTCGTGGAGAGTAAAGACACTGCTGAAGGAGAACATGACGATGCCCCAACTATATACACCGTCTTCCTTCCTCTCCTTGAGGGCCAGTTCCGTGCTGTTCTACAGGGCAATGAAAAGAATGAACTAGAGATTTGCCTCGAGAGTGGAGATAGTGCTGTTGAAACCAACCAAGGAAATTATCTTGTCTACATGCATGCTGGGACAAACCCCTTTGAAGTCATCAACCAGGCTGTAAAAGCCGTAGAAAAGCACATGCAAACTTTTCGTCATCGTGAGGAGAAAAAGTTGCCTTCTTTCATTGACTGGTTTGGCTGGTGTACATGGGATGCTTTTTACACTGATGTCACAGCTGAGGGTGTTGAAGAAGGCCTCAAAAGCTTGGCTGATGGAGGTACTCCATCACGGTTCCTTATCATAGATGACGGTTGGCAACAAATTGGTGGTGAAGCAAAGGACACCAATTGTGTTGTACAAGAAGGGGCACAGTTTGCTAACAGATTGACGGGAATAAAAGAGAACGAGAAATTCCAAAAGAATGGAAAAAGCAATGACCAGGTCCCAGGCCTGAAAATTGTTGTCGATGAATCCAAGCAACACCACAATGTGAAGTATGTGTATGTATGGCATGCTCTAGCCGGCTACTGGGGTGGGGTAAAGCCAGCAGCTGCTGGTATGGAGCATTATGACACCGCTTTGGCTTACCCAGTTCAGTCACCAGGTGTACTAGGCAACCAACCAGACATAGTCATGGACAGTCTTGCAGTACACGGCCTTGGTTTAGTGCACCCTAAAAAGGTCTTCAACTTCTACAACGAACTTCATGCCTACCTAGCTTCATGTGGAGTAGACGGTGTCAAAGTCGACGTGCAGAACATTATTGAAACTCTTGGTGCTGGCCACGGTGGCAGAGTTTCTCTCACTCGTGCCTATCATCAGGCCCTTGAGGCCTCCATTACTCGGAACTTCCAAGACAATGGTTGCATTGCTTGCATGTGTCATAATACTGATGGGATCTATAGTGCCAAGCAGACTGCTATAGTAAGAGCTTCTGACGATTTCTACCCTCGTGACCCTGCTTCTCACACCATCCACATCTCATCAGTTGCGTACAACTCTGTTTTCCTAGGAGAATTTATGCAACCCGATTGGGACATGTTTCATAGTTTGCACCCCGCCGCAGATTATCATGCTGCAGCTCGTGCTGTTGGAGGATGCCCAATTTATGTCAGTGATAAGCCTGGCAACCACAATTTTGAGCTTTTGAAGAAGCTTGTCCTTCCTGATGGATCAGTTCTTCGTGCCCAATTACCAGGCAGGCCAACCCGTGACTGTCTTTTTGTTGATCCGGCAAGAGATGGAATTAGCTTGCTTAAAATTTGGAATGTGAACAAATGCACCGGCGTGGTTGGCGTCTTCAACTGCCAAGGTGCTGGTTGGTGCAAGGTTGCAAAAAAGACCCGTATCCACGATGAATCTCCCGGCACCCTCACAGGTTCTGTGCAAGCCACCGATGTTGATTCCCTCTCTCAAGTTGCAGAGCCAGATTGGAATGGGGAAACGGTGGTTTATGCCTACAAATCAGGAGAAGTAATTCGGTTACCAAAAGGTGCCTCTTTACCTGTGACGCTGAAAGTTCTAGAATATGAACTCTTCCACTTCTGCCCTCTGAAGGAAATCACAGGGAACGTTTCCTTCGCACCAATAGGCTTGCTCGACATGTTCAACACTGGCGGTGCTTTGGAAGAGTTTGAAGTTCAAAATGAAAACCAATCTCCATCTGCGACAATTTTACTCAAAGTGAGGGGATGTGGCCGGTTTGGTGCTTACTCTTCACAGCGCCCACTGAAATGCCAAGTGGGTGGTGTTGAGACCGACTTTGAGTATGAACCTGGAACCGGATTGTTGACTTTTATTGTTCCGGTTGCAGAAGAGGAGATGTATAGATGGGGGATTGAAATCCAAGTTTGAAGTGGTAGTTTGTTTGTAGGGTTAGGAGAGATAAAATAAACTAGTGGTAGTGTATTAAAATGTCACCATTGTCAAGGGGAGTTTGTGGGTGACTTGATTTGGGGCAATGCTTCTTGTATTGCAGAGAGGGGGAGTCTCTCATGGGGGAGTGAGT

>CsRS-2

GTTTTGCCATTTGCCTGTGTTTTAAAATTTCTCCTTCGTTGCGAAGTTTGCATTCCTTTGTGGAATGCGCTTCTGCTTTTAGTTTGATTTCTATCTTCTCTCTGTCTCTCTATTCGTTGATCGGAGCTCGGGAAAAGATTTTCCGGCGAGGCGGCTACAGATACAGAGTTTATTGTGTAGCTAGCAAGAAGTAGGGTGCTGAAAGATGACGGTTACTCCTAATATCTCGGTCAGTGATGGAAACCTTGTGGTTCAGGGGAAGACCATACTGAAGGGTGTGCCCGAAAACATTGTTTTGACTCCGGGACCAGGCGTGGGCCTTGTAGCTGGTGCTTTCATTGGTGCCACTGCTGATCACAGCAAAAGCCTCCATGTCTTCCCCGTGGGCGTCTTAGAGGATCTCCGATTCATGTGTTGTTTTCGTTTCAAGCTATGGTGGATGACTCAGAGAATGGGGACATGTGGGAAGGACATTCCTCTTGAGACACAAATCTTGCTCGTGGAGAGTAAAGACACTGCTGAAGGAGAACATGACGATGCCCCAACTATATACACCGTCTTCCTTCCTCTCCTTGAGGGCCAGTTCCGTGCTGTTCTACAGGGCAATGAAAAGAATGAACTAGAGATTTGCCTCGAGAGTGGTGAGTAGATCATGGCAATCAAAAAGCATATGCTGTAGGTGCACATTTACCTAAGGCGTATATTCCGTTAAACCAGACCCATCCCAGCTGTCAAATAAAGAAGACTAACCATCCAATGTGGTGGGGTGGAGCTGTTGTTAATGTGAACACAACCAAGCCGGAGAAATGAATCAATCTCCCTACCATTCAAATGAATCAAACTAATGGAGTTTATAGAACTTGGGAAAGAAAAAGTAGGACCAACCGAGTTCAATAGAACCGAAACGACCTTGTGTTTTTGTGGTGTGATTTGATTTGCAGGAGATAGTGCTGTTGAAACCAACCAAGGAAATTATCTTGTCTACATGCATGCTGGGACAAACCCCTTTGAAGTCATCAACCAGGCTGTAAAAGCCGTAGAAAAGCACATGCAAACTTTTCGTCATCGTGAGGAGAAAAAGTTGCCTTCTTTCATTGACTGGTTTGGCTGGTGTACATGGGATGCTTTTTACACTGATGTCACAGCTGAGGGTGTTGAAGAAGGCCTCAAAAGCTTGGCTGATGGAGGTACTCCATCACGGTTCCTTATCATAGATGACGGTTGGCAACAAATTGGTGGTGAAGCAAAGGACACCAATTGTGTTGTACAAGAAGGGGCACAGTTTGCTAACAGATTGACGGGAATAAAAGAGAACGAGAAATTCCAAAAGAATGGAAAAAGCAATGACCAGGTCCCAGGCCTGAAAATTGTTGTCGATGAATCCAAGCAACACCACAATGTGAAGTATGTGTATGTATGGCATGCTCTAGCCGGCTACTGGGGTGGGGTAAAGCCAGCAGCTGCTGGTATGGAGCATTATGACACCGCTTTGGCTTACCCAGTTCAGTCACCAGGTGTACTAGGCAACCAACCAGACATAGTCATGGACAGTCTTGCAGTACACGGCCTTGGTTTAGTGCACCCTAAAAAGGTCTTCAACTTCTACAACGAACTTCATGCCTACCTAGCTTCATGTGGAGTAGACGGTGTCAAAGTCGACGTGCAGAACATTATTGAAACTCTTGGTGCTGGCCACGGTGGCAGAGTTTCTCTCACTCGTGCCTATCATCAGGCCCTTGAGGCCTCCATTACTCGGAACTTCCAAGACAATGGTTGCATTGCTTGCATGTGTCATAATACTGATGGGATCTATAGTGCCAAGCAGACTGCTATAGTAAGAGCTTCTGACGATTTCTACCCTCGTGACCCTGCTTCTCACACCATCCACATCTCATCAGTTGCGTACAACTCTGTTTTCCTAGGAGAATTTATGCAACCCGATTGGGACATGTTTCATAGTTTGCACCCCGCCGCAGATTATCATGCTGCAGCTCGTGCTGTTGGAGGATGCCCAATTTATGTCAGTGATAAGCCTGGCAACCACAATTTTGAGCTTTTGAAGAAGCTTGTCCTTCCTGATGGATCAGTTCTTCGTGCCCAATTACCAGGCAGGCCAACCCGTGACTGTCTTTTTGTTGATCCGGCAAGAGATGGAATTAGCTTGCTTAAAATTTGGAATGTGAACAAATGCACCGGCGTGGTTGGCGTCTTCAACTGCCAAGGTGCTGGTTGGTGCAAGGTTGCAAAAAAGACCCGTATCCACGATGAATCTCCCGGCACCCTCACAGGTTCTGTGCAAGCCACCGATGTTGATTCCCTCTCTCAAGTTGCAGAGCCAGATTGGAATGGGGAAACGGTGGTTTATGCCTACAAATCAGGAGAAGTAATTCGGTTACCAAAAGGTGCCTCTTTACCTGTGACGCTGAAAGTTCTAGAATATGAACTCTTCCACTTCTGCCCTCTGAAGGAAATCACAGGGAACGTTTCCTTCGCACCAATAGGCTTGCTCGACATGTTCAACACTGGCGGTGCTTTGGAAGAGTTTGAAGTTCAAAATGAAAACCAATCTCCATCTGCGACAATTTTACTCAAAGTGAGGGGATGTGGCCGGTTTGGTGCTTACTCTTCACAGCGCCCACTGAAATGCCAAGTGGGTGGTGTTGAGACCGACTTTGAGTATGAACCTGGAACCGGATTGTTGACTTTTATTGTTCCGGTTGCAGAAGAGGAGATGTATAGATGGGGGATTGAAATCCAAGTTTGAAGTGGTAGTTTGTTTGTAGGGTTAGGAGAGATAAAATAAACTAGTGGTAGTGTATTAAAATGTCACCATTGTCAAGGGGAGTTTGTGGGTGACTTGATTTGGGGCAATGCTTCTTGTATTGCAGAGAGGGGGAGTCTCTCATGGGGGAGTGAGT

>POD1-1

TATACTTCCACTTTTTTTGCACCATTCTTCACAACAACCCTATTCTCAGTCCTTTCTCAACTTCTAATACATAACAATGGATTCATCTTCTTCTTTCAAAGCCATTGTGACTTTGGCTTTCCTTTTTGTTTTCATGGGAAGCTCTTCAGCTCAACTTTCACCAACTTTCTATTCCTATTCTTGTCCCGATCTTTTTCCCACTGTGAAATCTGTAGTGCAATCTGCAATATCAAATGAAGCCCGGATGGGCGCTTCTCTTCTTCGATTGTTCTTCCATGACTGCTTTGTTAATGGTTGCGATGGATCAATCCTTCTTGATGACACATCTACGTTCATAGGGGAGAAGAGGGCAGCTCCAAACTTTAATTCTGTTAGGGGTTTTGATGTTGTTGACAATATAAAATCTGCAGTGGAAAATGTATGCCCTGGCGTAGTCTCATGTGCTGATGTGTTGGCCATTGCTTCCAGAGACTCTGTTGTTATTCTTGGAGGGCCTGACTGGAATGTAAAACTGGGAAGAAGAGATGCTAGGACAGCGAGCCAGGGTGCTGCCAATAGCAGCATTCCTCCTCCGACTTCTAACCTTACTGCCCTTGTCTCTAGTTTCAATGCTGTTGGCCTTTCCACCAAGGACGTGGTTACTCTATCTGGTGCTCACACAATTGGACAAGCAAGATGCACCTCCTTCAGAGCACGCATATATAATGAAACCAACATAGACAGTTCATTTGCTCAAACAAGGCGAGGCAACTGCCCGAGCACCTCTGGCTCAGGAGACAACAATTTGGCACCTCTTGATCTTCAAACCCCAACAGCTTTTGACAACAACTACTATAGAGACCTTGTCAACCAGAGAGGGCTCCTTCACTCTGATCAACAACTTTTCAATGGTGGATCAACCGACTCAATTGTGCAGACATATAGCAGCAGCCAAAGCACCTTCAACTTTGATTTTGTAGCGGCCATGATCAAGATGGGTGATATTAGCCCCCTCACCGGATCAAATGGAGAGATAAGGAAGAACTGTAGGAAGATTAATTAAAGGGCATGTTCATACGTATGAGCTTGAATTATTATTAAAGTGGTTGTTGACCTATTCTCATGATAGGGATGTTTGTTTTGATATAGTAAGTTTTGGTGCCTTGTGTGGGTATCAAGGCACTTGCCATGTCAAAGAATGCGTGCTTAGTTTAGTCTACAAGTGGACTTGATTGGACTTTCATATTTTTCCATAAATAAAATATTTAATAAGTTTTTGAACATTTGGTATTGCGAAGCGATTTTGTGATCCTTGATCTTACCAGCGATTGGGCCTCTTCATGATTTATCTTTTAGGTCCAGTGAGAGATTGAATTGAGCCTTGATCTCACTATGAGAGCTTGAAATTGGGCCCATGTTGTAATTCCAACAATTCTAACAATTTCGTAGCTCTCTTCTATCCATCGGACTAATTAAACATAGCC

>POD1-2

ATTCTCAGTCCTTTCTCAACTTCTAATACATAACAATGGATTCATCTTCTTCTTTCAAAGCCATTGTGACTTTGGCTTTCCTTTTTGTTTTCATGGGAAGCTCTTCAGCTCAACTTTCACCAACTTTCTATTCCTATTCTTGTCCCGATCTTTTTCCCACTGTGAAATCTGTAGTGCAATCTGCAATATCAAATGAAGCCCGGATGGGCGCTTCTCTTCTTCGATTGTTCTTCCATGACTGCTTTGTTAATGGTTGCGATGGATCAATCCTTCTTGATGACACATCTACGTTCATAGGGGAGAAGAGGGCAGCTCCAAACTTTAATTCTGTTAGGGGTTTTGATGTTGTTGACAATATAAAATCTGCAGTGGAAAATGTATGCCCTGGCGTAGTCTCATGTGCTGATGTGTTGGCCATTGCTTCCAGAGACTCTGTTGTTATTCTTGGAGGGCCTGACTGGAATGTAAAACTGGGAAGAAGAGATGCTAGGACAGCGAGCCAGGGTGCTGCCAATAGCAGCATTCCTCCTCCGACTTCTAACCTTACTGCCCTTGTCTCTAGTTTCAATGCTGTTGGCCTTTCCACCAAGGACGTGGTTACTCTATCTGGTTTACTCTACCGACCTTACTATAACATTTATTTTCATTTAATAATGTTGCAGAACAAGAAAAATTGGACAACTAGAAAATGACCAAAAGATTGAAGTGTGTGAACTAAATTCATTGTGACCTATAACAATCTAAATTTACTTTCGGACCACTTTATTGCCCTTTCTAAAATTATTTTCTGATCACTTTGTAGGCTATTTTTTCTACTCACTTGCATTTCCCTTTTTCATTTCATTTTTATCCAAATATTTAAACATTGTAGCACCTATTAATGGCAAATGATCGCAAGAGTGAACATTTTGAAACAACATTAATGCAAATTTTTTCCTTTTATTACGTCCAAATTCTAGATGGAAAACAAAATTCTTCCAAAACTTCTTTACAGAATTTCCACCACCAGATGTTGCATTTATTGTAAAGTGATAACCTACAACCCATCCATATCATTTTGTATAGTTTTTTCTTTTTTATGTTTTTTTGTTTGTATAGGCAAACTCATTCTAAAATATTTTTTCTACTTGGTTGTATGGCCTTACGAAAACAGGTGCTCACACAATTGGACAAGCAAGATGCACCTCCTTCAGAGCACGCATATATAATGAAACCAACATAGACAGTTCATTTGCTCAAACAAGGCGAGGCAACTGCCCGAGCACCTCTGGCTCAGGAGACAACAATTTGGCACCTCTTGATCTTCAAACCCCAACAGCTTTTGACAACAACTACTATAGAGACCTTGTCAACCAGAGAGGGCTCCTTCACTCTGATCAACAACTTTTCAATGGTGGATCAACCGACTCAATTGTGCAGACATATAGCAGCAGCCAAAGCACCTTCAACTTTGATTTTGTAGCGGCCATGATCAAGATGGGTGATATTAGCCCCCTCACCGGATCAAATGGAGAGATAAGGAAGAACTGTAGGAAGATTAATTAAAGGGCATGTTCATACGTATGAGCTTGAATTATTATTAAAGTGGTTGTTGACCTATTCTCATGATAGGGATGTTTGTTTTGATATAGTAAGTTTTGGTGCCTTGTGTGGGTATCAAGGCACTTGCCATGTCAAAGAATGCGTGCTTAGTTTAGTCTACAAGTGGACTTGATTGGACTTTCATA

>POD2-1

CACCCCCAAAAAACAAAAAAGTAGGAGAGGGCAGCGCGAAACAAAGATTAACACGATCGAATGGCCGAACGTCTCTCTCTCACTTCCGCATCTTCTTCTTCTTCTTCTTCGATGGCTGCTCTTAGCGCCGCCTCTCGCCTCCTCCCCTCCGCCACGAAAGCCGCCAGACTTTCTCTATCTACTCCCTCTCCCTCTCTCTCACCCTGTTCTTCCCTCAAATGCCTCCGATCATCTCGTCTTCTCTCTCCTCATCTCTTCCTTCGCCAGAGAAGGTCGGCGGTTCATGTGGCTGCTAGCAGTTTCGGAACAGTTGCGTCTCCGAAATGCTTTGCGTCCGATCCAGATCAACTGAAAAGTGCCAGAGAAGATATCAAGGAGCTTCTCAATACTAAGTTCTGCCATCCGATTCTGGTGCGTCTAGGATGGCATGATGCAGGTACTTATAACAAGAACATTGAGGAATGGCCACAAAGAGGTGGAGCTGATGGAAGTCTAAGGTTTGAAATTGAGCTGAAACATGCAGCTAACGCTGGTCTTGTAAATGCACTGAAACTTCTCGAGACTATCAAAGACAAATATTCTGGTGTGACATATGCGGATTTATTCCAATTGGCTAGTGCTACTGCAATCGAGGAGGCTGGCGGCCCCAAAATCCCTATGAAGTATGGAAGAGTAGATACTTCTAGTCCTGATCAATGTCCAGAAGAAGGAAGGCTTCCTGATGCGGGCCCCCCTAATCCTGCTGCTCATCTGCGAGAGGTTTTCTATAGGATGGGATTGAATGACAAGGAAATAGTTGCACTCTCGGGTGCGCACACACTGGGAAGGTCCAGACCAGAACGCAGTGGTTGGGGCAAGCCGGAGACAAAGTACACGAAAGATGGACCAGGAGCACCAGGAGGACAATCTTGGACAGCGCAATGGCTGAAGTTTGATAATTCCTACTTCAAGGATATCAAAGAAAGAATGGATGAAGATTTACTAGTTTTGCCAACCGATGCTGTTCTTTTTGAAGATCCAGCATTTAAGGTTTATGCTGAGAAATATGCTGCAGATCAGGATGAATTTTTCAAGGACTATGCTGAGGCCCATGCCAAACTCAGCAACCTTGGAGCCAAATTTGACCCTCCTGAGGGTATTTCAATAGATGATGGCCCTGCGACAGCTGCACCAGAGAAGTTTGTTGCCGCCAAGTACTCATCTGGAAAGGATTAAAATACCAGTATCTTGTTTTCTAGTGCCGTTTCTTTTAATAACACAAGATGAAGCTATTGTCAAACCCTGTGAACTCACATCTAAAACTTGGAAGTCTAAATGAAGGAGAGGAATAAATTCAATAGTGTTGTTGCCTATTTGAATTGCAGAGAGAACTATCGGACTCTATGAAGCAAAAGATTCGAGCAGAGTATGAAGCCATTGGGGGAAGCCCAGATAAGCCTCTCCAGTCAAACTATTTCCTTAATATTATCATTGTGATTGCGGTTTTGGCAATTTTGACATCTCTGTTTGGGAACTAA

>POD2-2

CACCCCCAAAAAACAAAAAAGTAGGAGAGGGCAGCGCGAAACAAAGATTAACACGATCGAATGGCCGAACGTCTCTCTCTCACTTCCGCATCTTCTTCTTCTTCTTCTTCGATGGCTGCTCTTAGCGCCGCCTCTCGCCTCCTCCCCTCCGCCACGAAAGCCGCCAGACTTTCTCTATCTACTCCCTCTCCCTCTCTCTCACCCTGTTCTTCCCTCAAATGCCTCCGATCATCTCGTCTTCTCTCTCCTCATCTCTTCCTTCGCCAGAGAAGGTCGGCGGTTCATGTGGCTGCTAGCAGTTTCGGAACAGTTGCGTCTCCGAAATGCTTTGCGTCCGATCCAGATCAACTGAAAAGTGCCAGAGAAGATATCAAGGAGCTTCTCAATACTAAGTTCTGCCATCCGATTCTGGTGCGTCTAGGATGGCATGATGCAGGTACTTATAACAAGAACATTGAGGAATGGCCACAAAGAGGTGGAGCTGATGGAAGTCTAAGGTTTGAAATTGAGCTGAAACATGCAGCTAACGCTGGTCTTGTAAATGCACTGAAACTTCTCGAGACTATCAAAGACAAATATTCTGGTGTGACATATGCGGATTTATTCCAATTGGCTAGTGCTACTGCAATCGAGGAGGCTGGCGGCCCCAAAATCCCTATGAAGTATGGAAGAGTAGATACTTCTAGTCCTGATCAATGTCCAGAAGAAGGAAGGCTTCCTGATGCGGGCCCCCCTAATCCTGCTGCTCATCTGCGAGAGGTTTTCTATAGGATGGGATTGAATGACAAGGAAATAGTTGCACTCTCGGGTGCGCACACACTGGGAAGGTCCAGACCAGAACGCAGTGGTTGGGGCAAGCCGGAGACAAAGTACACGAAAGATGGACCAGGAGCACCAGGAGGACAATCTTGGACAGCGCAATGGCTGAAGTTTGATAATTCCTACTTCAAGGATATCAAAGAAAGAATGGATGAAGATTTACTAGTTTTGCCAACCGATGCTGTTCTTTTTGAAGATCCAGCATTTAAGGTTTATGCTGAGAAATATGCTGCAGATCAGGATGAATTTTTCAAGGACTATGCTGAGGCCCATGCCAAACTCAGCAACCTTGGAGCCAAATTTGACCCTCCTGAGGGTATTTCAATAGATGATGGCCCTGCGACAGCTGCACCAGAGAAGTTTGTTGCCGCCAAGTACTCATCTGGAAAGAGAGAACTATCGGACTCTATGAAGCAAAAGATTCGAGCAGAGTATGAAGCCATTGGGGGAAGCCCAGATAAGCCTCTCCAGTCAAACTATTTCCTTAATATTATCATTGTGATTGCGGTTTTGGCAATTTTGACATCTCTGTTTGGGAACTAA

>SOD1-1

CAAAAACCAAACATTGGCCTGAAAAAAAATAGTAAAAGAACAGTCGCAAAGTATCCTCAACTTCAAGAAAACTTCAAGAAAGAGAAAATCAATTAGTTGCCTCAGTGATACTTCCTTAGCTGCAAGAAAGAGCTATAACGGAAATGGGTTGGTCATCCTCTTGTTGCTGCAATCTTTTCCCCACAAGCTCTCATATTCTTGGGGCTAGGGACTTGTCCAAGCTGTTGAAGAACCCCAAGCTTCCTCTTCGGAGTCAGCGGCAAAAGAGAGAACAGCATGGGACCCAAAAGACAACAAAAGTTCTTGCCTACCATGGCCTGCAAAAACCACCTTATAAACTTGATGCACTAGAACCATATATGAGCCAAAGGACACTTGAGATGCACTGGGGAGAGCATCATCGTCTTTATGTGGAAGGTCTGAACAAACAACTTGAAAAGAATGACATACTGTATGGCCACACCATGGATGAACTTGTCAAAGTGACATATAACAATGGGAATCCATTACCAGAATTTAATAACGCTGCCCAGGTTTGGAATCATGACTTCTTTTGGGAATCGATGCAACCAGGAGGTGGTAACATGCCCATAATGGGTCTTCTTCAGCAGATTGAAAAGGATTTTGCTTCTTTTAATAACTTCAAAGAGAAGTTCATAGAAGCAGCCCTCACATTATTTGGCTCTGGCTGGGTTTGGCTTGTATTGAAGAGAAGTGAGAAACGACTTGAAGTGGTTAGAACATCAAATGCCGTCAGCCCTCTTGTGTGGGATGACATTCCCATTATCAGTTTGGATATGTGGGAACATGCTTATTATTTGGATTATAAGAACGACAAAGGTAGGTACGTTAATGTCTTCATGAACCACCTTGTGTCTTGGAATGCGGCAATGGGACGCATGGCTCGTGCACAGGCATTTGTGAATTTAGGCGAACCCAAAATTCCTATTGCTTGAAATGCTTCTCTCTCTCTCTCTCTCTCTCTACACAAGCATGAGGTGTCTCAAATCTGAACACTCATAAAATTCACGTCTTTCGCAAGTCATACGTGACCTTATCAGTATAAGTGTTTGAGTATGAAACAAAGAAGCTCCCATGCATAGCCTGGCAACCAATCTCCAACTACCTTAAATGCCCCACTAAAGCTGCTGTAGTATTTGTAGCAAAAATCAGAGAATCCTTGTTATACATGTTTTGAGGCTTATTCATGTTAGATTTGTCGTTTAAACTATGTACTCGCACATTTTCTAATTAATTTAATTTTTGTATGCTTTGGTGTCATGTGGATTGTATTTGTCTATTACAAATTTGGAGTGAGAAAAGTTATGTATGGCATGTAGACG

>SOD1-2

CAAAAACCAAACATTGGCCTGAAAAAAAATAGTAAAAGAACAGTCGCAAAGTATCCTCAACTTCAAGAAAACTTCAAGAAAGAGAAAATCAATTAGTTGCCTCAGGTTTGTAGATATGCACGTATCTATCTGCTTATTTACACGCCTTTTCATTGAAGCAGGCCATGTGTTTGTTGAAATGCCCAGCAGGGAAGAAAAAAAAAAAAAGAGAGCTTTTATTTATCTTTTTGTGCTTTGTTTGGATCTTGGATTTGAATTTGTCAAGGAATTTGTGGAGATAAATGAAAGGGAAGAGAAAGTGGGGAAAAAAAAAAAAAAAACTTATACTTCATTAACAATTCAAGATCCAAACACAAGAAAAAAAGTTTGATGTAGAGGAATTGAGGTCCTAAATCTCATTGGTTATGCAGTGATACTTCCTTAGCTGCAAGAAAGAGCTATAACGGAAATGGGTTGGTCATCCTCTTGTTGCTGCAATCTTTTCCCCACAAGCTCTCATATTCTTGGGGCTAGGGACTTGTCCAAGCTGTTGAAGAACCCCAAGCTTCCTCTTCGGAGTCAGCGGCAAAAGAGAGAACAGCATGGGACCCAAAAGACAACAAAAGTTCTTGCCTACCATGGCCTGCAAAAACCACCTTATAAACTTGATGCACTAGAACCATATATGAGCCAAAGGACACTTGAGATGCACTGGGGAGAGCATCATCGTCTTTATGTGGAAGGTCTGAACAAACAACTTGAAAAGAATGACATACTGTATGGCCACACCATGGATGAACTTGTCAAAGTGACATATAACAATGGGAATCCATTACCAGAATTTAATAACGCTGCCCAGGTTTGGAATCATGACTTCTTTTGGGAATCGATGCAACCAGGAGGTGGTAACATGCCCATAATGGGTCTTCTTCAGCAGATTGAAAAGGATTTTGCTTCTTTTAATAACTTCAAAGAGAAGTTCATAGAAGCAGCCCTCACATTATTTGGCTCTGGCTGGGTTTGGCTTGTATTGAAGAGAAGTGAGAAACGACTTGAAGTGGTTAGAACATCAAATGCCGTCAGCCCTCTTGTGTGGGATGACATTCCCATTATCAGTTTGGATATGTGGGAACATGCTTATTATTTGGATTATAAGAACGACAAAGGTAGGTACGTTAATGTCTTCATGAACCACCTTGTGTCTTGGAATGCGGCAATGGGACGCATGGCTCGTGCACAGGCATTTGTGAATTTAGGCGAACCCAAAATTCCTATTGCTTGAAATGCTTCTCTCTCTCTCTCTCTCTCTCTACACAAGCATGAGGTGTCTCAAATCTGAACACTCATAAAATTCACGTCTTTCGCAAGTCATACGTGACCTTATCAGTATAAGTGTTTGAGTATGAAACAAAGAAGCTCCCATGCATAGCCTGGCAACCAATCTCCAACTACCTTAAATGCCCCACTAAAGCTGCTGTAGTATTTGTAGCAAAAATCAGAGAATCCTTGTTATACATGTTTTGAGGCTTATTCATGTTAGATTTGTCGTTTAAACTATGTACTCGCACATTTTCTAATTAATTTAATTTTTGTATGCTTTGGTGTCATGTGGATTGTATTTGTCTATTACAAATTTGGAGTGAGAAAAGTTATGTATGGCATGTAGACG

>bHLH-1

AACCCACGTGTTCAAATATCATTGGATCCATCCTTTTTTGGTGCTTGGTATCTTGATTCGTGTGATTCCGCTTTTCTCATCATTCTCCCTTTTGTTTTTTTCTAATCAAATCATCAAATTCAAAAAAGGAGGCTTCAATTTTCACCTCCAAAAAGTCCCAACAGATCAGATCAGATCATCACCTATTTTGATGAATCGGATTAATCAAAACTCTTAATTTTAGGTTTTTGATTTGATTCACGGCCCAACCTTGCAATTACTTGGTTTCTGAGTTTCGATTCACAACCCTAACCTTGCGATTGCTCGGTTTCTGAACCAATTATGAACTCGTTTGATGATAACAGTTGGGATCTACTCGATTATGGCAGCTTCATCGACGATGTTACATCGACCGATCTCTATTGGGGCAGCCAAAGTGCTGGTGTGAAGGCTGATGTTTCACTTCCTGGGGCCACACCGCAAGATGAGGAATGCACAGAGAAAGAATGCCATAGGAAGAGGTTTGTAGAATTGAGCTCTATCTTGGAACCTGGGAGACCTCCCAAAACTGATAAATTGGCCATACTCGGTGATGCCATCCGTGTTTTGAACCAGCTGAGAGCTGAATCTCAGGAGTTCAAAGAGACAAATGAGAAGCTCTTAGAAGAGATAAAAAGTTTAAAGGCTGAGAAGAATGAACTTCGTGATGAAAAACTCGTATTGAAGGGGGACAAAGAAAGGATGGAGCAGCAGCTGAAAGCTATGCCTGTCTCTCCTGCTGCTTTTATGCCGCCCCATCATCATCCAGCAGCAGCATATCATGCCGGGACAAACAAGATTCCTGTTTTTCCCAGTTACGGGTTTGTTCCGATGTGGCAATATCTCCCTCCATCTGCCATTGATACGACGAAGGATGAAGAGCTCAGGCCTCCAGCTGCTTAGAACTAGTTGCAAATATTTGCACCAATTCCGGATGCTTTATTTTTTATGATTTCATGGATTTTTTAGCCATTTCAACTTGTAAATATTGCTGCTCTATTGTTCTATTCTAATTTGACCAATTCTTCAATATTCTGTGATTGGTAGGCTGAATTGGAAGAAATTAATAGTTGGATTAGATTCGCTTTGTGTTTGGAACTTTTGAAAGACTTTGAATTCTGGAATTGAATTTCTTCACAAAAATCTACTAATATTTTAAATTCAATGGACTTTGATGTGCAACTAAAATTTGATTGTTCAAGGTCAGACATGTATTCAGTGCATGTATTTTTG

>bHLH-2

CTTTTTTGGTGCTTGGTATCTTGATTCGTGTGATTCCGCTTTTCTCATCATTCTCCCTTTTGTTTTTTTCTAATCAAATCATCAAATTCAAAAAAGGAGGCTTCAATTTTCACCTCCAAAAAGTCCCAACAGATCAGATCAGATCATCACCTATTTTGATGAATCGGATTAATCAAAACTCTTAATTTTAGGTTTTTGATTTGATTCACGGCCCAACCTTGCAATTACTTGGTTTCTGAGTTTCGATTCACAACCCTAACCTTGCGATTGCTCGGTTTCTGAACCAATTATGAACTCGTTTGATGATAACAGTTGGGATCTACTCGATTATGGCAGCTTCATCGACGATGTTACATCGACCGATCTCTATTGGGGCAGCCAAAGTGCTGGTGTGAAGGCTGATGTTTCACTTCCTGGGGCCACACCGCAAGATGAGGAATGCACAGAGAAAGAATGCCATAGGAAGAGGACACGGAATGCTTCATGCAGCAAACCAGGCACGAAAGCTTGTCGTGAGAGATTACGGAGAGAAAGATTGAACGACAGGTTTGTAGAATTGAGCTCTATCTTGGAACCTGGGAGACCTCCCAAAACTGATAAATTGGCCATACTCGGTGATGCCATCCGTGTTTTGAACCAGCTGAGAGCTGAATCTCAGGAGTTCAAAGAGACAAATGAGAAGCTCTTAGAAGAGATAAAAAGTTTAAAGGCTGAGAAGAATGAACTTCGTGATGAAAAACTCGTATTGAAGGGGGACAAAGAAAGGATGGAGCAGCAGCTGAAAGCTATGCCTGTCTCTCCTGCTGCTTTTATGCCGCCCCATCATCATCCAGCAGCAGCATATCATGCCGGGACAAACAAGATTCCTGTTTTTCCCAGTTACGGGTTTGTTCCGATGTGGCAATATCTCCCTCCATCTGCCATTGATACGACGAAGGATGAAGAGCTCAGGCCTCCAGCTGCTTAGAACTAGTTGCAAATATTTGCACCAATTCCGGATGCTTTATTTTTTATGATTTCATGGATTTTTTAGCCATTTCAACTTGTAAATATTGCTGCTCTATTGTTCTATTCTAATTTGACCAATTCTTCAATATTCTGTGATTGGTAGGCTGAATTGGAAGAAATTAATAGTTGGATTAGAT

The sequencing results:

>COR-1

AGGGTCGGAACAGGAGAGCGCACGAGGGAGCTTCCAGGGGGAAACGCCTGGTATCTTATAGTCCTGTCGGGTTTCGCCACCTCTGACTTGAGCGTCGATTTTGTGATGCTCGTCAGGGGGGCGGAGCCTATGGAAAAACGCCAGCAACGCGGCCTTTTTACGGTTCCTGGCCTTTTGCTGGCCTTTTGCTCACATGTTCTTTCCTGCGTTATCCCCTGATTCTGTGGATAACCGTATTACCGCCTTTGAGTGAGCTGATACCGCTCGCCGCAGCCGAACGACCGAGCGCAGCGAGTCAGTGAGCGAGGAAGCGGAAGAGCGCCCAATACGCAAACCGCCTCTCCCCGCGCGTTGGCCGATTCATTAATGCAGCTGGCACGACAGGTTTCCCGACTGGAAAGCGGGCAGTGAGCGCAACGCAATTAATGTGAGTTAGCTCACTCATTAGGCACCCCAGGCTTTACACTTTATGCTTCCGGCTCGTATGTTGTGTGGAATTGTGAGCGGATAACAATTTCACACAGGAAACAGCTATGACCATGATTACGCCAAGCTTGGTACCGAGCTCGGATCCACTAGTAACGGCCGCCAGTGTGCTGGAATTGCCCTTATTTGGCAATGAAGACGACGGGTTCAACAACTTCAACGGAGTTGATAAGTTCTGATCTGCAAGATCTGGGGAACGCCGCAAAGAAGTTCGCAAGTCATGCCATCATGCTCACTTCTGGCCTCGGTCTGGGCTCTGTTCTTCTTCAATGGATAGCTTCAATCGCTGCTATTTATTTGTTGGTTTTGGATCGAACAAACTGGAGGACCAACATTCTTACCGCACTTTTAATCCCATACATTTTCTTCAGTCTTCCTTCATTAGTGTTCAGCTTGCTCAGTGGAGAGAAAGGGCAATTCTGCAGATATCCATCACACTGGCGGCCGCTCGAGCATGCATCTAGAGG

>COR-2

GCTTTTGCTGGCCTTTTGCTCACATGTTCTTTCCTGCGTTATCCCCTGATTCTGTGGATAACCGTATTACCGCCTTTGAGTGAGCTGATACCGCTCGCCGCAGCCGAACGACCGAGCGCAGCGAGTCAGTGAGCGAGGAAGCGGAAGAGCGCCCAATACGCAAACCGCCTCTCCCCGCGCGTTGGCCGATTCATTAATGCAGCTGGCACGACAGGTTTCCCGACTGGAAAGCGGGCAGTGAGCGCAACGCAATTAATGTGAGTTAGCTCACTCATTAGGCACCCCAGGCTTTACACTTTATGCTTCCGGCTCGTATGTTGTGTGGAATTGTGAGCGGATAACAATTTCACACAGGAAACAGCTATGACCATGATTACGCCAAGCTTGGTACCGAGCTCGGATCCACTAGTAACGGCCGCCAGTGTGCTGGAATTGCCCTTGATTATCTTTGCTACTTTGGCTTCATACTTTAATTTCTTTTTTGCTGCTTTTAATCTGTCTGACAATGCAGTGGAGGGATTGGAAAATGGATTGCTTTCATCGCTGTGGTATTGCGTCTCTTCTTCCCCAAACATTTTCCTGATTGGCTAGAAATGCCAGGAGCACTGATTCTCCTCATCGTGGTAGCTCCAAGCTTGATTGCTGATACACTGAGGGACAGTTTTATCGGTGCTGTAATATGTCTTGCCATCGCATGTTACTTGCTTCAAGAACACATCCGAGCGTCAGGTGGGTTCAGAAATTCCTTCACGAAGAGTAGTGGTGTCTCCAACTCCGTTGGCATAATCATTCTTCTAAGGGCAATTCTGCAGATATCCATCACACTGGCGGCCGCTCGAGCATGCATCTAGAGGGCCCAATCGCCCTA

>SUS-1

CTTGCAGTCAGACAGAGCCTGGTTTTGGAATTTTTAAGGTGAACGCCAATAGTCTAGAAGTGGATTCGATACAGCCAAAGACTACTTGAAGCTCAAAGAAATAGTGTACGATGAAAATTGGGCAATGGATAAAAATGCACTGGAAATAGATTTGGAGCATGTGACTTCTCTACTCCTCGCCTAACCCTTTCTTCTTCTATTGGAAATGGAGTTGACTTCATGTCAAAGTTCATGACCTCAAGGATTAGTGGTGATCTTGAACGTGCAAAGCCTATGCTTGAGTACTTACTTGCCCTAGATCATCACGGAGAGAATCTTATGATCAATGAGAATATCAACACAGTTTCCAAGCTTCAGGCAGGCTTGATCGTGGCTGATGTTTACGTTTCTGCCCTCCCAAAAAACACTCCTTACCAAAATTTTGAGCAGAAGCTTAAAGAGTGGGGGTTCGAGAAAGGGTGGGGAGAAAACGCAGACAGAGTTAAAGAGACAATGAAGATCCTTTCAGAGATACTACAAGCACCGGACCCAATTAAAATGGAATCGTTCTTCAGAAGGCTTCCAAATATATTCAAAATCGTGATCTTCTCAGTCCATGGCTACTTTGGCCAGTCTGATGTCCTTGGCTTGCCTGATACTGGTGGCCAGGTTGTTTACATTCTAGACCAGGTGAAAGCTTTAGAGGAGGAATTGCTGCTGAGAATTAAGCAGCAAGGACTGAGTGTGAAGCCTCAGATTCTTGTGGTGACTCGTCTCATACCAGATGCAAAAGGGACTAAGTGCAGCGAGGAGATTGAGCCTGTCCTCAACACGACACACTCCCACATCCTTAGAGTCCCATTCAAGACAGACAAAGGGGTTCTGCACCAATGGGATGCTACTTAAGGGCAATTCTGCAGATATCCATCACACTGGCGGCCGCTCGAGCATGCATCTAGAGGGC

>SUS-2

TTGGGCCCTCTAGATGCATGCTCGAGCGGCCGCCAGTGTGATGGATATCTGCAGAATTGCCCTTGCTTTTCTGGGTTTGTAGGAATGGGGAGAAGGTTGATGAAACCCCACCAAATAATGGAAGTAATAGACAAAGCAATCGAGGACAAGCGTGAAAGAGCCAGAGTTTTGGAGGGTTTACTCGGCCAAGTCTTCAGTTCTACCCAGGAGGTGGCTATAAATCCACCTTACGTAGCCTTGGCAGTCAGACAGAGCCCTGGTTTTTGGGAATTTTTTAAGGTGAACGCCAATAGTCTAGAAGTGGATTCGATAACAGCCAAAGACTACTTGAAGCTCAAAGAAATAGTGTACGGTGAAAATTGGGCAATGGATAAAAATGCACTGGAAATAGATTTTGGAGCATGTGACTTCTCTACTCCTCGCCTAACCCTTTCTTCTTCTATTGGAAATGGAGTTGACTTCATGTCAAAGTTCATGACCTCAAGGATTAGTGGTGATCTTGAACGTGCAAAGCCTTTGCTTGAGTACTTACTTGCCCTAGATCGTCACGGAGAGAATCTTATGATCAATGAGAATATCAACACAGTTTCCAAGCTTCAGGCAGGCTTGATCGTGGCTGATGTTTACGTTTCTGCCCTCCCAAAAAACACTCCTTACCAAAATTTTGAGCAGAAGCTTAAAGAGTGGGGGTTCGAGAAAGGGTGGGGAGAAAACGCAGACAGAGTTAAAGAGACAATGAAGATCCTTTCAGAGATACTACAAGCACCGGACCCAATTAAAATGGAATCGTTCTTCAGAAGGCTTCCAAATATATTCAAAATCGTGATCTTCTCAGTCCATGGCTACTTTGGCCAGTCTAATGTCCTTGGCTTGCCTGATACTGGTGGCCAGGTTGTTTACATTCTAGACCAGGTGAAAGCTTTAGAGGAGGAATTGCTGCTGAGAATTAAGCAGCAAGGACTGAGTGTGAAGCCTCAGATTCTTGTGGTGACTCGTCTCATACCAGATGCAAAAGGGACTAAGTGCAGCGAGGAGATTGAGCCTGTCCTCAACACGACACACTCCCACATCCTTAGAGTCCCATTCAAGACAGACAAAGGGGTTCTGCACCAATGGGTATCCCGGTTTGATGACTACCCTTACCTTGAGAGATTAAGGGCAATTCCAGCACACTGGCGGCCGTTACTAGTGGATCCGAGCTG

>SUS-3

GCTCGCCGCAGCCGAACGACCGAGCGCAGCGAGTCAGTGAGCGAGGAAGCGGAAGAGCGCCCAATACGCAAACCGCCTCTCCCCGCGCGTTGCCGATTCATTAATGCAGCTGGCACGACAGGTTTCCCGACTGGAAAGCGGGCAGTGAGCGCAACGCAATTAATGTGAGTTAGCTCACTCATTAGGCACCCCAGGCTTTACACTTTATGCTTCCGGCTCGTATGTTGTGTGGAATTGTGAGCGGATAACAATTTCACACAGGAAACAGCTATGACCATGATTACGCCAAGCTTGGTACCGAGCTCGGATCCACTAGTAACGGCCGCCAGTGTGCTGGAATTGCCCTTATCTCATATATTGTGCAAACCTATTTTTGTATTTTCAGGCTTAAAGAGTGGGGGTTCGAGAAAGGGTGGGGAGAAAACGCAGACAGAGTTAAAGAGACAATGAAGATCCTTTCAGAGATACTACAAGCACCGGACCCAATTAAAATGGAATCGTTCTTCAGAAGGCTTCCAAATATATTCAAAATCGTGATCTTCTCAGTCCATGGCTACTTTGGCCAGTCTGATGTCCTTGGCTTGCCTGATACTGGTGGCCAGGTTGTTTACATTCTAGACCAGGTGAAAGCTTTAGAGGAGGAATTGCTGCTGAGAATTAAGCAGCAAGGACTGAGTGTGAAGCCTCAGATTCTTGTGGTGACTCGTCTCATACCAGATGCAAAAGGGACTAAGTGCAGCGAGGAGATTGAGCCTGTCCTCAACACGACACACTCCCACATCCTTAGAGTCCCATTCAAGACAGACAAAGGGGTTCTGCACCAATGGGTATCCCGGTTTGATGACTACCCTTACCTTGAGAGAAAGGGCAATTCTGCAGATATCCATCACACTGGCGGCCGCTCGAGCATGCATCG

>SUS-4

ACGCAATTAATGTGAGTAGCTCACTCATTAGGCACCCCAGGCTTACACTTATGCTTCGGCTCGTATGTTGTGTGGAATTGTGAGCGGATACAATTCACACAGGAAACAGCTATGACCATGATTACGCCAAGCTTGGTACCGAGCTCGGATCCACTAGTAACGGCCGCCAGTGTGCTGGAATTGCCCTTATCTCATATATTGTGCAAACCTATTTTTGTATTTTCAGGCTTAAAGAGTGGGGGTTCGAGAAAGGGTGGGGAGAAAACGCAGACAGAGTTAAAGAGACAATGAAGATCCTTTCAGAGATACTACAAGCACCGGACCCAATTAAAATGGAATCGTTCTTCAGAAGGCTTCCAAATATATTCAAAATCGTGATCTTCTCAGTCCATGGCTACTTTGGCCAGTCTGATGTCCTTGGCTTGCCTGATACTGGTGGCCAGGTATATAACACAACCAGTCACATTAGTATGAGCAATAGTACATACATACAAAGCTCGATGTACAACCAGGACTAACAGGCTTTTGAATTTTCAACAGGTTGTTTACATTCTAGACCAGGTGAAAGCTTTAGAGGAGGAATTGCTGCTGAGAATTAAGCAGCAAGGACTGAGTGTGAAGCCTCAGATTCTTGTGGTGAGCGATGCATAAAATTCACCAAGAAATGATTTAATTGACCGAGAGAAGATAGGTTTAAGAGTTTATAAGTCTTTCTTGCTTTGCTTATATGTCCACAGGTGACTCGTCTCATACCAGATGCAAAAGGGACTAAGTGCAGCGAGGAGATTGAGCCTGTCCTCAACACGACACACTCCCACATCCTTAGAGTCCCATTCAAGACAGACAAAGGGGTTCTGCACCAATGGGTATCCCGGTTTGATGACTACCCTTACCTTGAGAGAAAGGGCAATTCTGCAGATATCCATCACACTGGCGGCCGCTCGAG

>RS-1

GATCCTATAGGGCGAATTGGGCCCTCTAGATGCATGCTCGAGCGGCCGCCAGTGTGATGGATATCTGCAGAATTGCCCTTCTCCTAATATCTCGGTCAGTGATGGAAACCTTGTGGTTCAGGGGAAGACCATACTGAAGGGTGTGCCCGAAAACATTGTTTTGACTCCGGGACCAGGCGTGGGCCTTGTAGCTGGTGCTTTCATTGGTGCCACTGCTGATCACAGCAAAAGCCTCCATGTCTTCCCCGTGGGCGTCTTAGAGGATCTCCGATTCATGTGTTGTTTTCGTTTCAAGCTATGGTGGATGACTCAGAGAATGGGGACATGTGGGAAGGACATTCCTCTTGAGACACAAATCTTGCTCGTGGAGAGTAAAGACACTGCTGAAGGAGAACATGACGATGCCCCAACTATATACACCGTCTTCCTTCCTCTCCTTGAGGGCCAGTTCCGTGCTGTTCTACAGGGCAATGAAAAGAATGAACTAGAGATTTGCCTCGAGAGTGGAGATAGTGCTGTTGAAACCAACCAAGGAAATTATCTTGTCTACATGCATGCTGGGACAAACCCCTTTGAAGTCATCAACCAGGCTGTAAAAGCCGTAGAAAAGCACATGCAAGGGCAATTCCAGCACACTGGCGGCCGTTACTAGTGGATCCGAGCTCGGTACCAAGCTTGGCGTAATCATGGTCATAGCTGTTTCCTGTGTGAAATTGTTATCCGCTCACAATTCCACACAACATACGAGCCGGAAGCATAAAGTGTAAAGCCTGGGGGTGCCTAATGAGTGAGCTAACTCACATTAATTGCGTTGCGCTCA

>RS-2

TATAGGGCGATTGGGCCCTCTAGATGCATGCTCGAGCGGCCGCCAGTGTGATGGATATCTGCAGAATTGCCCTTCTCCTAATATCTCGGTCAGTGATGGAAACCTTGTGGTTCAGGGGAAGACCATACTGAAGGGTGTGCCCGAAAACATTGTTTTGACTCCGGGACCAGGCGTGGGCCTTGTAGCTGGTGCTTTCATTGGTGCCACTGCTGATCACAGCAAAAGCCTCCATGTCTTCCCCGTGGGCGTCTTAGAGGATCTCCGATTCATGTGTTGTTTTCGTTTCAAGCTATGGTGGATGACTCAGAGAATGGAGACATGTGGGAAGGACATTCCTCTTGAGACACAAATCTTGCTCGTGGAGAGTAAAGACACTGCTGAAGGAGAACATGACGATGCCCCAACTATATACACCGTCTTCCTTCCTCTCCTTGAGGGCCAGTTCCGTGCTGTTCTACAGGGCAATGAAAAGAATGAACTAGAGATTTGCCTCGAGAGTGGTGAGTAGATCATGGCAATCAAAAAGCATATGCTGTAGGTGCACATTTACCTAAGGCGTATATTCCGTTAAACCAGACCCATCCCAGCTGTCAAATAAAGAAGACTAACCATCCAATGTGGTGGGGTGGAGCTGTTGTTAATGTGAACACAACCAAGCCGGAGAAATGAATCAATCTCCCTACCATTCAAATGAATCAAACTAATGGAGTTTATAGAACTTGGGAAAGAAAAAGTAGGACCAACCGAGTTCAATAGAACCGAAACGACCTTGTGTTTTTGTGGTGTGATTTGATTTGCAGGAGATAGTGCTGTTG

>POD1-1

TACGCAAACCGCCTCTCCCCGCGCGTTGCCGATTCATTAATGCAGCTGGCACGACAGGTTTCCCGACTGGAAAGCGGGCAGTGAGCGCAACGCAATTAATGTGAGTTAGCTCACTCATTAGGCACCCCAGGCTTTACACTTTATGCTTCCGGCTCGTATGTTGTGTGGAATTGTGAGCGGATAACAATTTCACACAGGAAACAGCTATGACCATGATTACGCCAAGCTTGGTACCGAGCTCGGATCCACTAGTAACGGCCGCCAGTGTGCTGGAATTGCCCTTTCCCACTGTGAAATCTGTAGTGCAATCTGCAATATCAAATGAAGCCCGGATGGGCGCTTCTCTTCTTCGATTGTTCTTCCATGACTGCTTTGTTAATGGTTGCGATGGATCAATCCTTCTTGATGACACATCTACGTTCATAGGGGAGAAGAGGGCAGCTCCAAACTTTAATTCTGTTAGGGGTTTTGATGTTGTTGACAATATAAAATCTGCAGTGGAAAATGTATGCCCTGGCGTAGTCTCATGTGCTGATGTGTTGGCCATTGCTTCCAGAGACTCTGTTGTTATTCTTGGAGGGCCTGACTGGAATGTAAAACTGGGAAGAAGAGATGCTAGGACAGCGAGCCAGGGTGCTGCCAATAGCAGCATTCCTCCTCCGACTTCTAACCTTACTGCCCTTGTCTCTAGTTTTAATGCTGTTGGCCTTTCCACCAAGGACGTGGTTACTCTATCTGGTGCTCACACAAGGGCAATTCTGCAGATATCCATCACACTGGCGGCCGCTCGAGCATGCATCTAGAGGGCCCAATCGCCCTATATTAGTCAAC

>POD1-2

CAGCTATGACCATGATTACGCCAAGCTTGGTACCGAGCTCGGATCCACTAGTACGCCCGCCAGTGTGCTGGAATTGCCCTTTAACCTACAACCCATCCATATCATTTTGTATAGTTTTTTCTTTTTTATGTTTTTTTGTTTGTATAGGCAAACTCATTCTAGAATATTTTTTCTACTTGGTCGTATGGCCTTACGAAAACAGGTGCTCACACAATTGGACAAGCAAGATGCACCTCCTTCAGAGCACGCATATATAATGAAACCAACATAGACAGTTCATTTGCTCAAACAAGGCGAGGCAACTGCCCGAGCACCTCTGGCTCAGGAGACAACAATTTGGCACCTCTTGATCTTCAAACCCCAACAGCTTTTGACAACAACTACTATAGAGACCTTGTCAACCAGAGAGGGCTCCTTCACTCTGATCAACAACTTTTCAATGGTGGATCAACCGACTCAATTGTGCAGACATATAGCAGCAGCCAAAGCACCTTCAACTTTGATTTTGTAGCGGCCATGATCAAGATGGGTGATATTAGCCCCCTCACCGGATCAAATGGAGAGATAAGGAAGAACTGTAGGAAGATTAATTAAAGGGCATGTTCATACGTATGAGCTTGAATTATTATTAAAGTGGTTGTTGACCTATTCTCATGATAGGGATGTTTGCTTTGATATAGTAAGTTTTGGTGCCTTGTGTGGGTATCAAGGCACTTGCCATGTCAAAGAATGCGTGCTTAGTTTAGTCTACAAGTGGACTTGATTGGACTTTCAAAGGGCAATTCTGCAGATATCCATCACACTGGCGGCCGCTCGAGCATGCATCTAGAGGGCCCAATCGCCCTATAGAAGTTCATCA

>POD2-1

CGGACAGGTATCCGGTAAGCGGCAGGGTCGGAACAGGAGAGCGCACGAGGGAGCTTCCAGGGGGAAACGCCTGGTATCTTTATAGTCCTGTCGGGTTTCGCCACCTCTGACTTGAGCGTCGATTTTTGTGATGCTCGTCAGGGGGGCGGAGCCTATGGAAAAACGCCAGCAACGCGGCCTTTTTACGGTTCCTGGCCTTTTGCTGGCCTTTTGCTCACATGTTCTTTCCTGCGTTATCCCCTGATTCTGTGGATAACCGTATTACCGCCTTTGAGTGAGCTGATACCGCTCGCCGCAGCCGAACGACCGAGCGCAGCGAGTCAGTGAGCGAGGAAGCGGAAGAGCGCCCAATACGCAAACCGCCTCTCCCCGCGCGTTGGCCGATTCATTAATGCAGCTGGCACGACAGGTTTCCCGACTGGAAAGCGGGCAGTGAGCGCAACGCAATTAATGTGAGTTAGCTCACTCATTAGGCACCCCAGGCTTTACACTTTATGCTTCCGGCTCGTATGTTGTGTGGAATTGTGAGCGGATAACAATTTCACACAGGAAACAGCTATGACCATGATTACGCCAAGCTTGGTACCGAGCTCGGATCCACTAGTAACGGCCGCCAGTGTGCTGGAATTGCCCTTAGCTATTGTCAAACCCTGTGAACTCACATCTAAAACTTGGAAGTCTAAATGAAGGAGAGGAATAAATTCAATAGTGTTGTTGCCTATTTGAATTGCAGAGAGAACTATCGGACTCTATGAAGCAAAAGATTCGAGCAGAGTATGAAGCCATTAAGGGCAATTCTGCAGATATCCATCACACTGGCGGCCGCTCGAGCATGCATCTAGAGGGCCCAATCGCCT

>POD2-2

TCTTCCTGCGTTATCCCCTGATTCTGTGGATAACCGTATTACCGCCTTTGAGTGAGCTGATACCGCTCGCCGCAGCCGAACGACCGAGCGCAGCGAGTCAGTGAGCGAGGAAGCGGAAGAGCGCCCAATACGCAAACCGCCTCTCCCCGCGCGTTGGCCGATTCATTAATGCAGCTGGCACGACAGGTTTCCCGACTGGAAAGCGGGCAGTGAGCGCAACGCAATTAATGTGAGTTAGCTCACTCATTAGGCACCCCAGGCTTTACACTTTATGCTTCCGGCTCGTATGTTGTGTGGAATTGTGAGCGGATAACAATTTCACACAGGAAACAGCTATGACCATGATTACGCCAAGCTTGGTACCGAGCTCGGATCCACTAGTAACGGCCGCCAGTGTGCTGGAATTGCCCTTAGCACCAGGAGGACAATCTTGGACAGCGCAATGGCTGAAGTTTGATAATTCCTACTTCAAGGATATCAAAGAAAGAATGGATGAAGATTTACTAGTTTTGCCAACCGATGCTGTTCTTTTTGAAGATCCAGCATTTAAGGTTTATGCTGAGAAATATGCTGCAGATCAGGATGAATTTTTCAAGGACTATGCTGAGGCCCATGCCAAACTCAGCAACCTTGGAGCCAAATTTGACCCTCCTGAGGGTATCTCAATAGATGATGGCCCTGCGACAGCTGCACCAGAGAAGTTTGTTGCCGCCAAGTACTCATCTGGAAAGAGAGAACTATAAGGGCAATTCTGCAGATATCCATCACACTGGCGGCCGCTCGAGCATGCATCTAGAGGGCCCAATCGCCCTATATAT

>SOD-1

GGGATGACTCTATAGGGCGATTGGGCCCTCTAGATGCATGCTCGAGCGGCCGCCAGTGTGATGGATATCTGCAGAATTGCCCTTCAATTAGTTGCCTCAGTGATACTTCCTTAGCTGCAAGAAAGAGCTATAACGGAAATGGGTTGGTCATCCTCTTGTTGCTGCAATCTTTTCCCCACAAGCTCTCATATTCTTGGGGCTAGGGACTTGTCCAAGCTGTTGAAGAACCCCAAACTTCCTCTTCGGAGTCAGCGGCAAAAGAGAGAACAGCATGGGACCCAAAAGACACCAAAAGTTCTTGCCTACCATGGCCTGCAAAAACCACCTTATAAACTTGATGCACTAGAACCATATATGAGCCAAAGGACACTTGAGATGCACTGGGGAGAGCATCATCGTCTTTATGTGGAAGGTCTGAACAAACAACTTGAAAAGAATGACATACTGTATGGCCACACCATGGATGAACTTGTCAAAGTGACATATAACAATGGGAATCCATTACCAGAATTTAATAACGCTGCCCAGGTTTGGAATCATGACTTCTTTTGGGAATCGATGCAACCAGGAGGTGGTAACATGCCCATAATGGGTCTTCTTCAGCAGATTGAAAAGGATTTTGCTTCTTTTAATAACTTCAAAGAGAAGTTCATAGAAGCAGCCCTCACATTATTTGGCTCTGGCTGGGTTTGGCTTGTATTAAGGGCAATTCCAGCACACTGGCGGCCGTTACTAGTGGATCCGAGCTCGGTACCAAGCTTGGCGTAATCATGGTCATAGCTGTTTCCTGTGTGAAATTGTTATCCGCTCACAATTCCACACAACATACGAGCCGGAAGCATAAAGTGTAAGCCTGGGGTGCCTAATGAGTGAGCTAACTCACATTAATTGCGTTGCGCTCACTGCCCGCTTTCCAGTCGGAAACCTGTCGTGCCAGCTGCATTAATGAATCGGCCACGCGCGGGGAGAAGGCGGTTGCGTATTGGGCGCTCTCGCTCTCGCTCACTGACTCGCTGCGCTCGTCGTCGGCTGCGCGAGCGGTATCAGCTCACTCAAGGCGGTAATACGGTTATCCACAGAATCAG

>SOD-2

GGGAGAACTATATAGGGCGATTGGGCCCTCTAGATGCATGCTCGAGCGGCCGCCAGTGTGATGGATATCTGCAGAATTGCCCTTAATGCCCAGCAGGGAAGAAAAAAAAAAAAAGAGAGCTTTTATTTATCTTTTTGTGCTTTGTTTGGATCTTGGATTTGAATTTGTCAAGGAATTTGTGGAGATAAATGAAAGGGAAGAGAAAGTGGGAAAAAAAAAAAAAAAAAACTTATACTTCATTAACAATTCAAGATCCAAACACAAGAAAAAAAGTTTGATGTAGAGGAATTGAGGTCCTAAATCTCATTGGTTATGCAGTGATACTTCCTTAGCTGCAAGAAAGAGCTATAACGGAAATGGGTTGGTCATCCTCTTGTTGCTGCAATCTTTTCCCCACAAGCTCTCATATTCTTGGGGCTAGGGACTTGTCCAAGCTGTTGAAGAACCCCAAACTTCCTCTTCGGAGTCAGCGGCAAAAGAGAGAACAGCATGGGACCCAAAAGACACCAAAAGTTCTTGCCTACCATGGCCTGCAAAAAAGGGCAATTCCAGCACACTGGCGGCCGTTACTAGTGGATCCGAGCTCGGTACCAAGCTTGGCGTAATCATGGTCATAGCTGTTTCCTGTGTGAAATTGTTATCCGCTCACAATTCCACACAACATACGAGCCGGAAGCATAAAGTGTAAAGCCTGGGGTGCCTAATGAGTGAGCTAACTCACATTAATTGCGTTGCGCTCACTGCCCGCTTTCCAGTCGGGAAACCTGTCGTGCCAGCTGCATTAATGAATCGGCCACGCGCGGGGAGAAGGCGGTTTGCGTATTGGGCGCTCTTCCGCTTCCTCGCTCACTGACTCGCTGCGCTCGGTCGTTCGGCTGCGGCGAGCGTATCAGCTCACTCAAAGGCGGTAATACGGTATCCACAGAATCAGGGATAACGCAGAAAGAACATGTGAGCAAAGCAGCAAAGCAGACCGTAAAAGCCGGGTTGCTGCGTTTTCATAGCTCCGCCCCTGACGAGCATCCAAAATCGACGCTCAAGTCAGAGTGCGAAGCGGAACGACTATAAGAAACCAGCGTCCCTGGAGCTCCTCGTGGCTCTCTGTCGACTGCGCTTACGATAC

>bHLH-1

TGAGCGAGGAAGCGGAAGAGCGCCAATACGCAAACCGCCTCTCCCCGCGCGTTGCCCGATTCATTAATGCAGCTGGCACGACAGGTTTCCCGACTGGAAAGCGGGCAGTGAGCGCAACGCAATTAATGTGAGTTAGCTCACTCATTAGGCACCCCAGGCTTTACACTTTATGCTTCCGGCTCGTATGTTGTGTGGAATTGTGAGCGGATAACAATTTCACACAGGAAACAGCTATGACCATGATTACGCCAAGCTTGGTACCGAGCTCGGATCCACTAGTAACGGCCGCCAGTGTGCTGGAATTGCCCTTCCATAGGAAGAGGTTTGTAGAATTGAGCTCTATCTTGGAACCTGGGAGACCTCCCAAAACTGATAAATTGGCCATACTCGGTGATGCCATCCGTGTTTTGAACCAGCTGAGAGCTGAATCTCAGGAGTTCAAAGAGACAAATGAGAAGCTCTTAGAAGAGATAAAAAGTTTAAAGGCTGAGAAGAATGAACTTCGTGATGAAAAACTCGTATTGAAGGGGGACAAAGAAAGGATGGAGCAGCAGCTGAAAGCTATGCCTGTCTCTCCTGCTGCTTTTATGCCGCCCCATCATCATCCAGCAGCAGCATATCATGCCGGGACAAACAAGATTCCTGTTTTTCCCAGTTACGGGTTTGTTCCGATGTGGCAATATCTCCCTCCATCTGCCATTGATACGACGAAGGATGAAGAGCTCAGGCCTCCAGCTGCTTAGAACTAGTTGCAAATATTTGCACCAATTCCGGATGCTTTATTTTTTATGATTTCATGGATTTTTTAGCCATTTCAACTTGTAAAGGGCAATTCTGCAGATATCCATCACACTGGCGGCCGCTCGAGCATGCATCTAGAGGGCCCAATCGCCC

>bHLH-2

GGCCCTCTAGATGCATGCTCGAGCGGCCGCCAGTGTGATGGATATCTGCAGAATTGCCCTTGGAATGCTTCATGCAGCAAACCAGGCACGAAAGCTTGTCGTGAGAGATTACGGAGAGAAAGATTGAACGACAGGTTTGTAGAATTGAGCTCTATCTTGGAACCTGGGAGACCTCCCAAAACTGATAAATTGGCCATACTCGGTGATGCCATCCGTGTTTTGAACCAGCTGAGAGCTGAATCTCAGGAGTTCAAAGAGACAAATGAGAAGCTCTTAGAAGAGATAAAAAGTTTAAAGGCTGAGAAGAATGAACTTCGTGATGAAAAACTCGTATTGAAGGGGGACAAAGAAAGGATGGAGCAGAAGGGCAATTCCAGCACACTGGCGGCCGTTACTAGTGGATCCGAGCTCGGTACCAAGCTTGGCGTAATCATGGTCATAGCTGTTTCCTGTGTGAAATTGTTATCCGCTCACAATTCCACACAACATACGAGCCGGAAGCATAAAGTGTAAAGCCTGGGGTGCCTAATGAGTGAGCTAACTCACATTAATTGCGTTGCGCTCACTGCCCGCTTTCCAGTCGGGAAACCTGTCGTGCCAGCTGCATTAATGAATCGGCCAACGCGCGGGGAGAGGCGGTTTGCGTATTGGGCGCTCTTCCGCTTCCTCGCTCACTGACTCGCTGCGCTCGGTCGTTCGGCTGCGGCGAGCGGTATCAGCTCACTCAAAGGCGGTAATACGGTTATCCACAGAATCAGGGGATAACGCAGGAAAGAACATGTGAGCAAAAGGGCAGCAAAAGGGCAGGAACCGTAAAAAGGCCGCGTTGCTGGCGTTTTTCCATAGGCTCCGCCCCCCTGACGAGCATCACAAAAATCGACGCTCAAGTCAGAGGTGGCGAAC
