# Supplementary figures and images for: Comprehensive profiling of alternative splicing landscape during cold acclimation in tea plant
Source: BMC Genomics. 2020 Jan 20;21:65. doi: 10.1186/s12864-020-6491-6 (PMC6971990; doi:10.1186/s12864-020-6491-6)

A

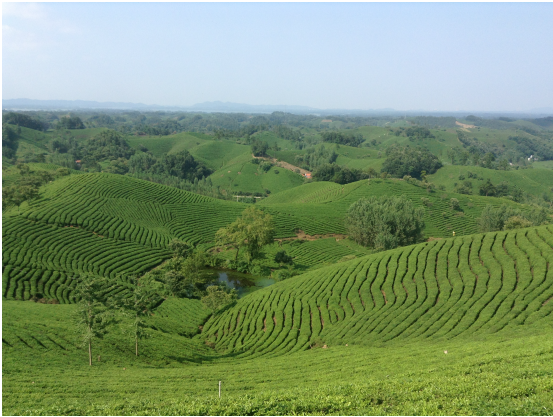

Tea plantation

B

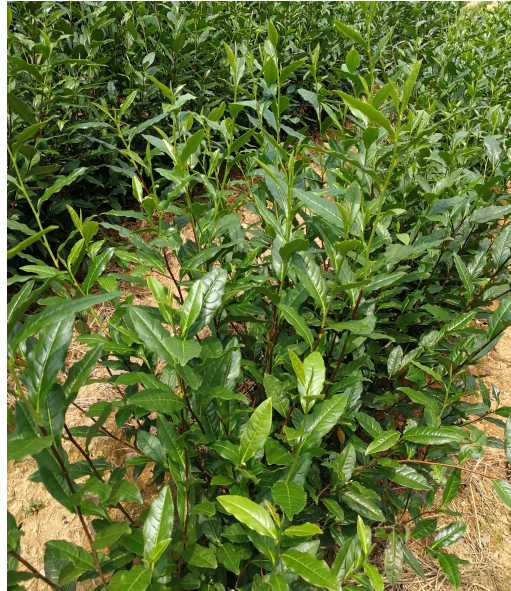

Tea plants

Supplement: Supplementary file 9 — Additional file 9: Figure S1. The picture of tea plantation and tea plants. [file 12864_2020_6491_MOESM9_ESM.pdf]

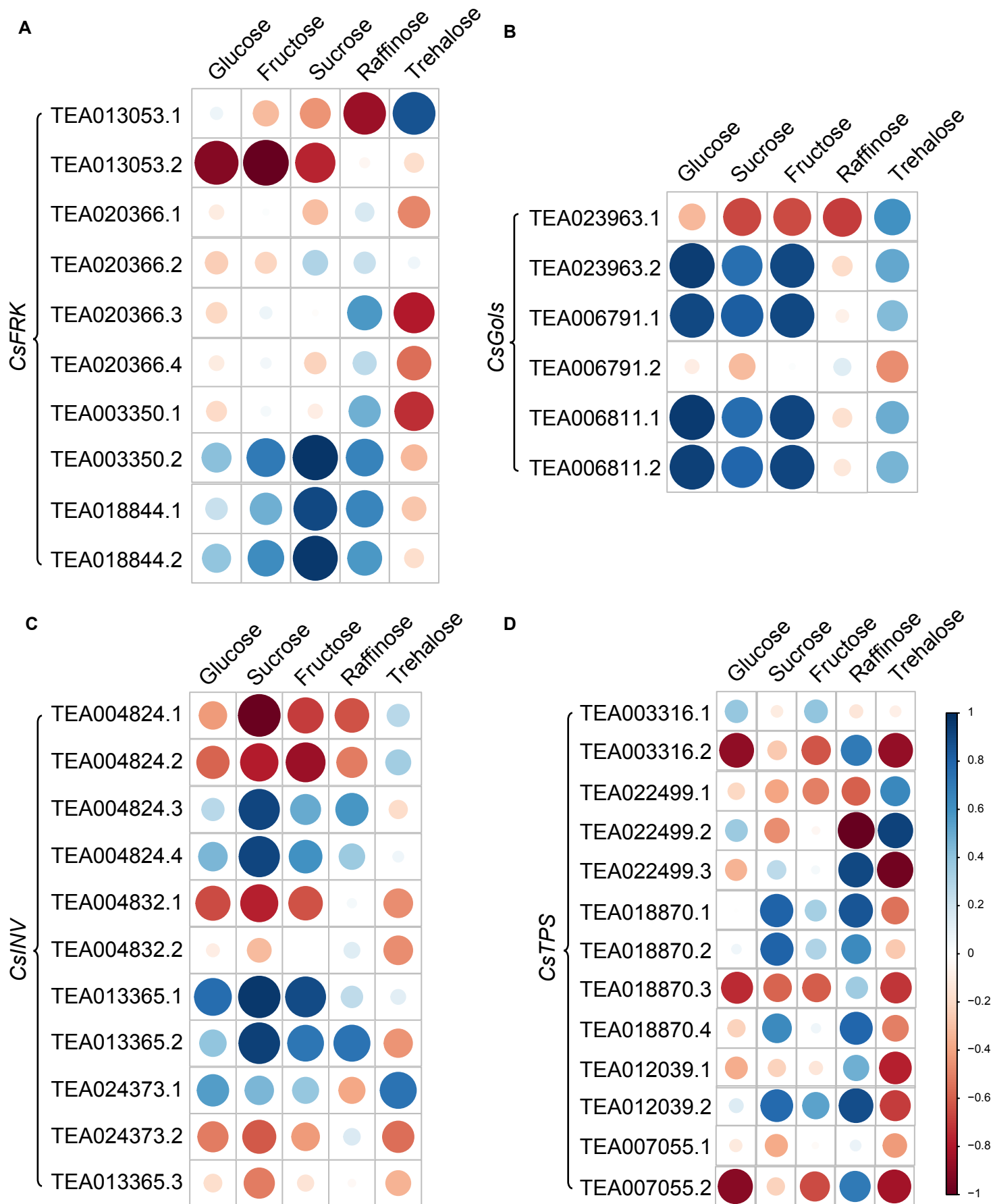

Supplement: Supplementary file 12 — Additional file 12: Figure S4. Correlation analysis of AS transcriptions and sugar content. [file 12864_2020_6491_MOESM12_ESM.pdf]

conformity:

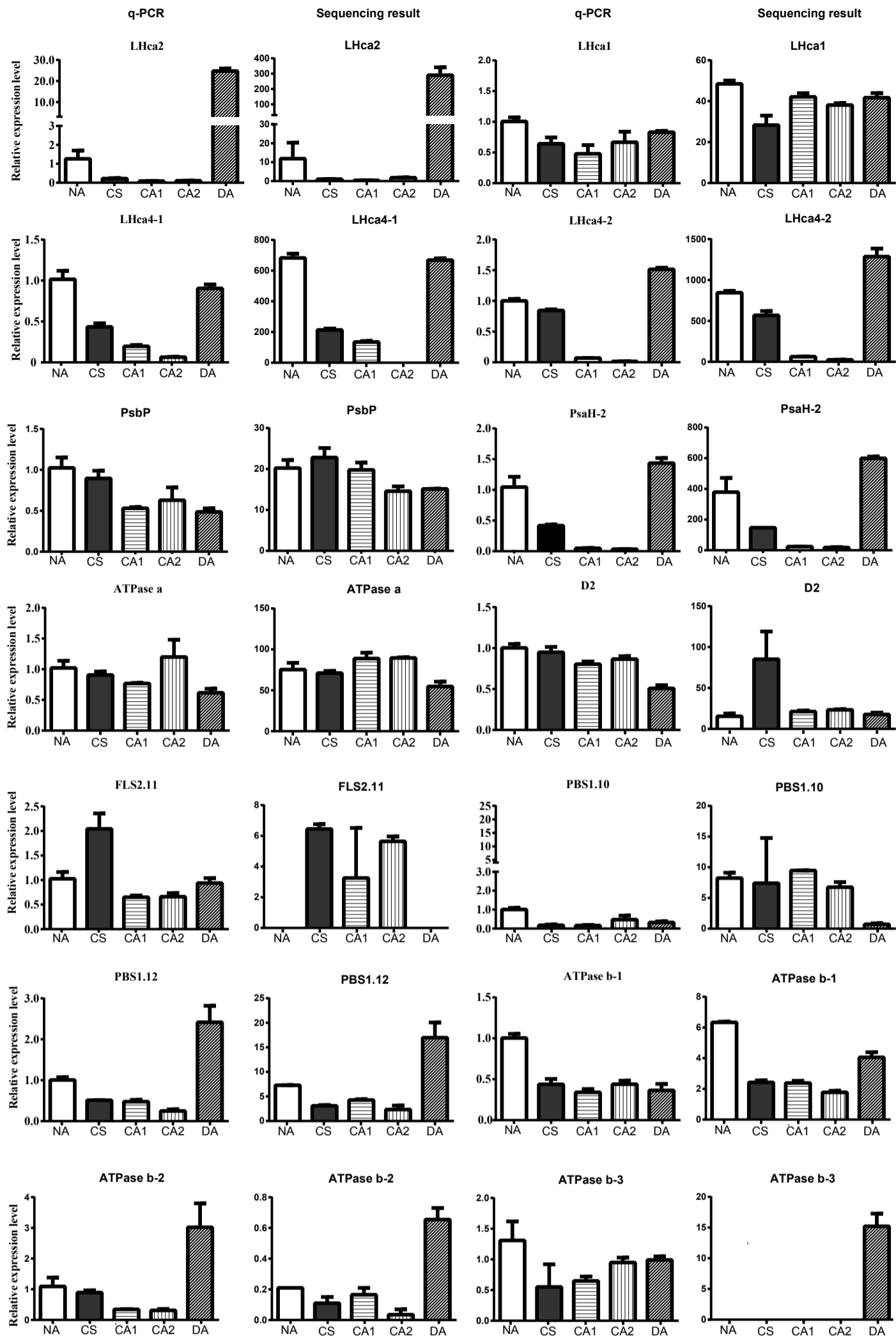

inconformity:

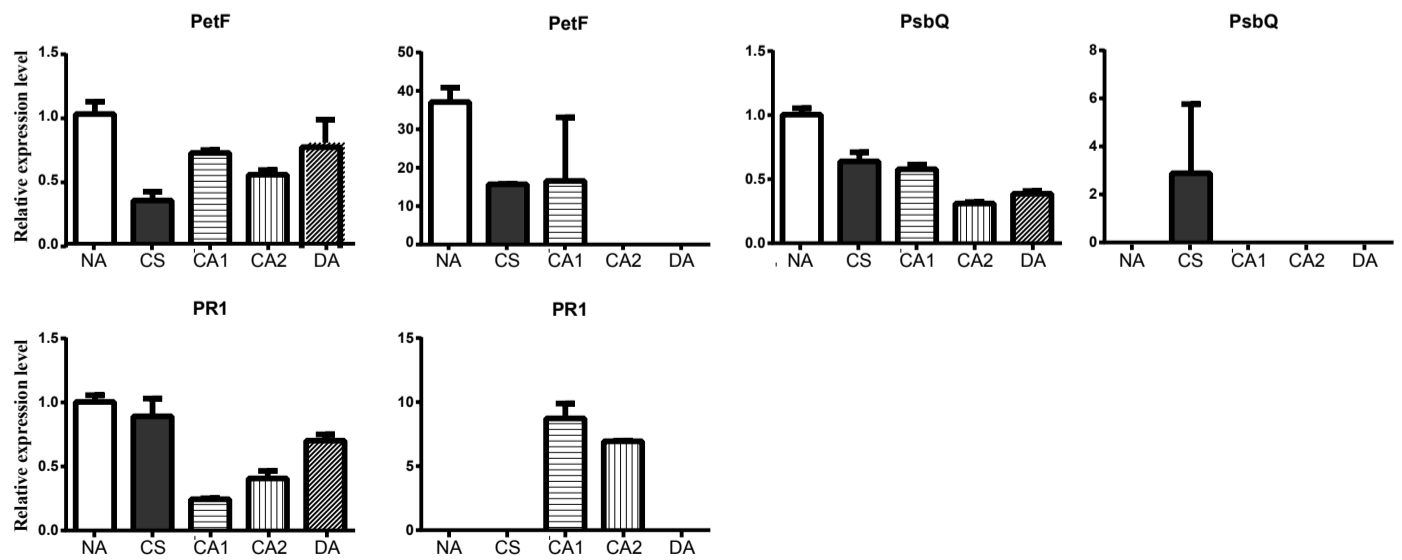

Supplement: Supplementary file 14 — Additional file 14: Figure S6. Expression analysis of qRT-PCR and sequencing results. [file 12864_2020_6491_MOESM14_ESM.pdf]

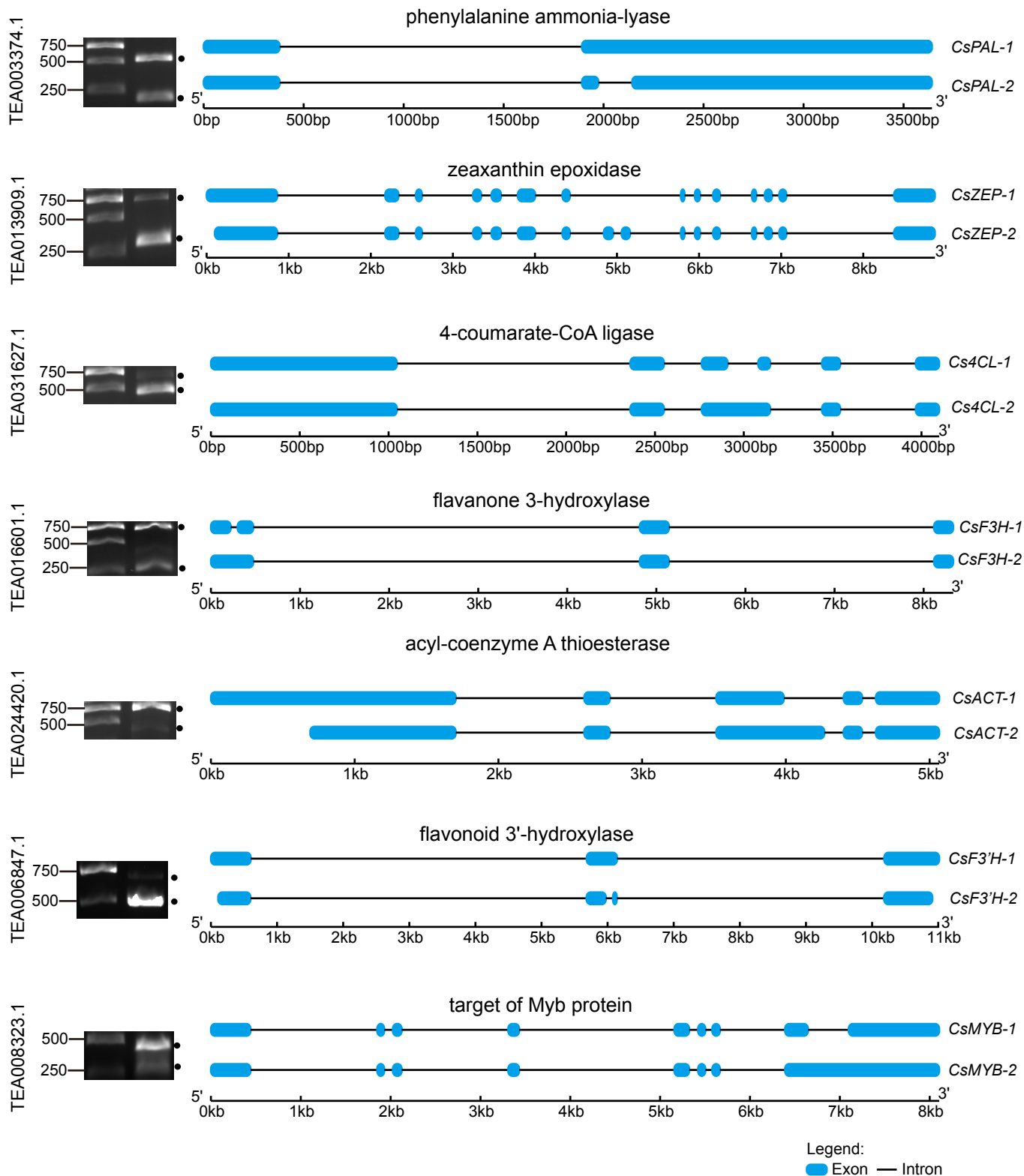

Supplement: Supplementary file 15 — Additional file 15: Figure S7. Verification of alternatively spliced isoforms. [file 12864_2020_6491_MOESM15_ESM.pdf]
