# Supplementary material for: Comprehensive profiling of alternative splicing landscape during cold acclimation in tea plant
Source: BMC Genomics. 2020 Jan 20;21:65. doi: 10.1186/s12864-020-6491-6 (PMC6971990; doi:10.1186/s12864-020-6491-6)
Supplement: Supplementary file 10 — Additional file 10: Figure S2. Percentages of four main AS types during cold acclimation. [file 12864_2020_6491_MOESM10_ESM.pdf]

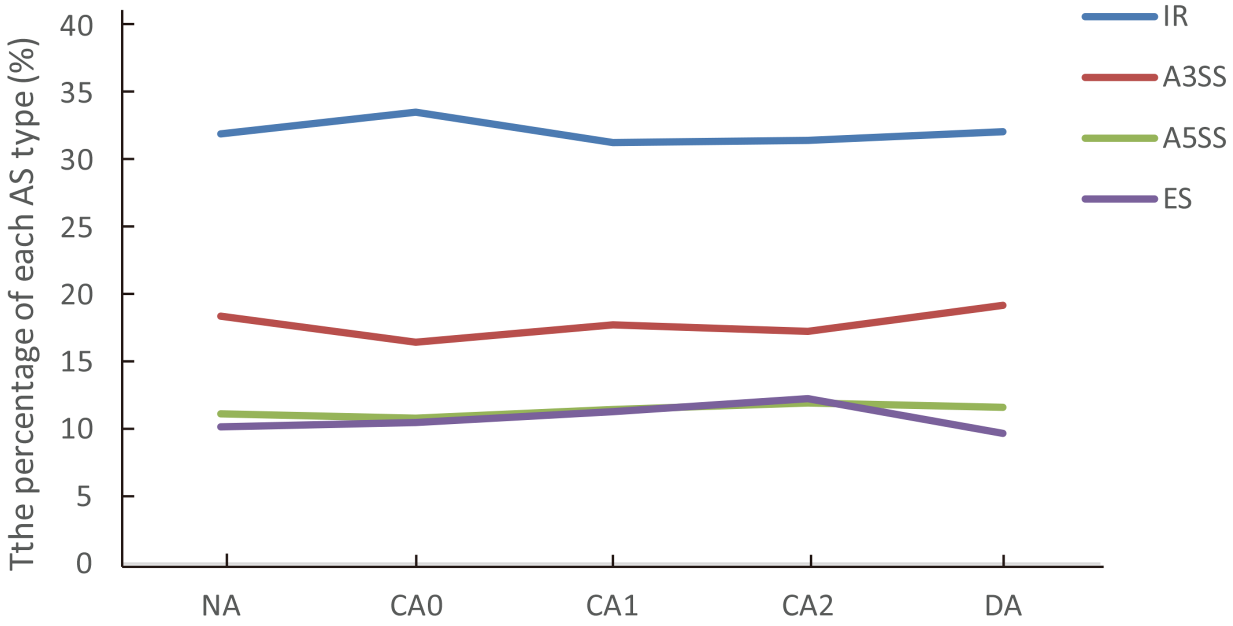

Figure S1. The percentage of the four main AS types during cold acclimation. IR: intron retention; ES: exon skipping; A3SS: alternative 3' splice site; A5SS alternative 5' splice site. NA: non-acclimation; CA0: cold stress of 6 hours at 10˚C, day/night; CA1: cold acclimation of 7 days at 10/4˚C,day/night; CA2: cold acclimation of 7 days at 4/0˚C, day/night; DA: de-acclimation of 7 days at 25/20˚C, day/night.
