# Supplementary material for: Comprehensive profiling of alternative splicing landscape during cold acclimation in tea plant
Source: BMC Genomics. 2020 Jan 20;21:65. doi: 10.1186/s12864-020-6491-6 (PMC6971990; doi:10.1186/s12864-020-6491-6)
Supplement: Supplementary file 11 — Additional file 11: Figure S3. Expression analysis of AS transcripts during cold acclimation. [file 12864_2020_6491_MOESM11_ESM.pdf]

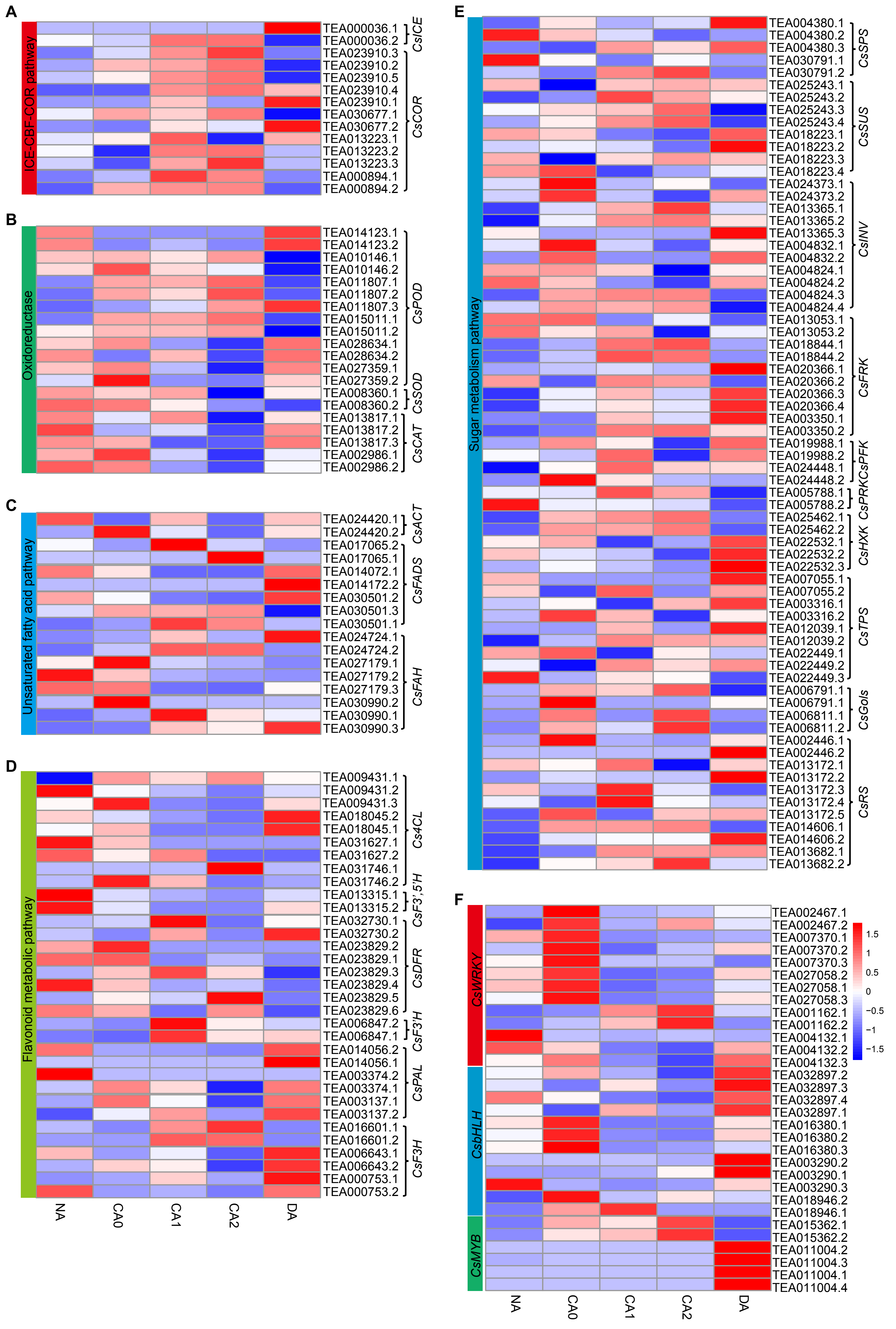

Figure S3. Expression analysis of cold stress-responsive AS transcripts during cold acclimation. The relative transcript accumulation is shown in the heat map generated with R software. Blue and red bars indicate lower and higher expression levels, respectively. Different types of transcripts are colored with red, blue and green on the left. The ID and name of the genes are indicated on the right. *CsICE*: inducer of CBF expression gene; *CsCOR*: cold regulated gene; *CsSUS*: sucrose synthase gene; *CsRS*: raffinose synthase gene; *CsTPS*: trehalose-6-phosphate synthase gene; *CsFRK*: fructokinase gene; *CsPFK*: 6-phosphate fructokinase gene; *CsINV*: invertase gene; *CsHXK*: hexokinase gene; *CsGols*: galactinol synthase gene; *CsCAT*: catalase gene; *CsSOD*: superoxide dismutase gene; *CsPOD*: peroxidase gene; *CsACT*: acyl-coenzyme A thioesterase; *CsFADS*: fatty acid desaturase; *CsFAH*: fatty acid hydrolase; *Cs4CL*: 4-coumarate--CoA ligase; *CsF3',5'H*: flavonoid-3',5'-hydroxylase; *CsDFR*:dihydroflavonol 4 -reductase;*CsF3'H*: flavonoid 3'-hydroxylase; *CsPAL*: phenylalanine ammonia-lyase; *CsF3H*: flavanone 3-hydroxylase. NA: non-acclimation; CA0: cold stress of 6 hours at 10˚C, day/night; CA1: cold acclimation of 7 days at 10/4˚C, day/night; CA2: cold acclimation of 7 days at 4/0˚C, day/night; DA: de-acclimation of 7 days at 25/20˚C, day/night.
