# Supplementary material for: Comprehensive profiling of alternative splicing landscape during cold acclimation in tea plant
Source: BMC Genomics. 2020 Jan 20;21:65. doi: 10.1186/s12864-020-6491-6 (PMC6971990; doi:10.1186/s12864-020-6491-6)
Supplement: Supplementary file 13 — Additional file 13: Figure S5. Domain analysis of SUS and RS in AS transcripts. [file 12864_2020_6491_MOESM13_ESM.pdf]

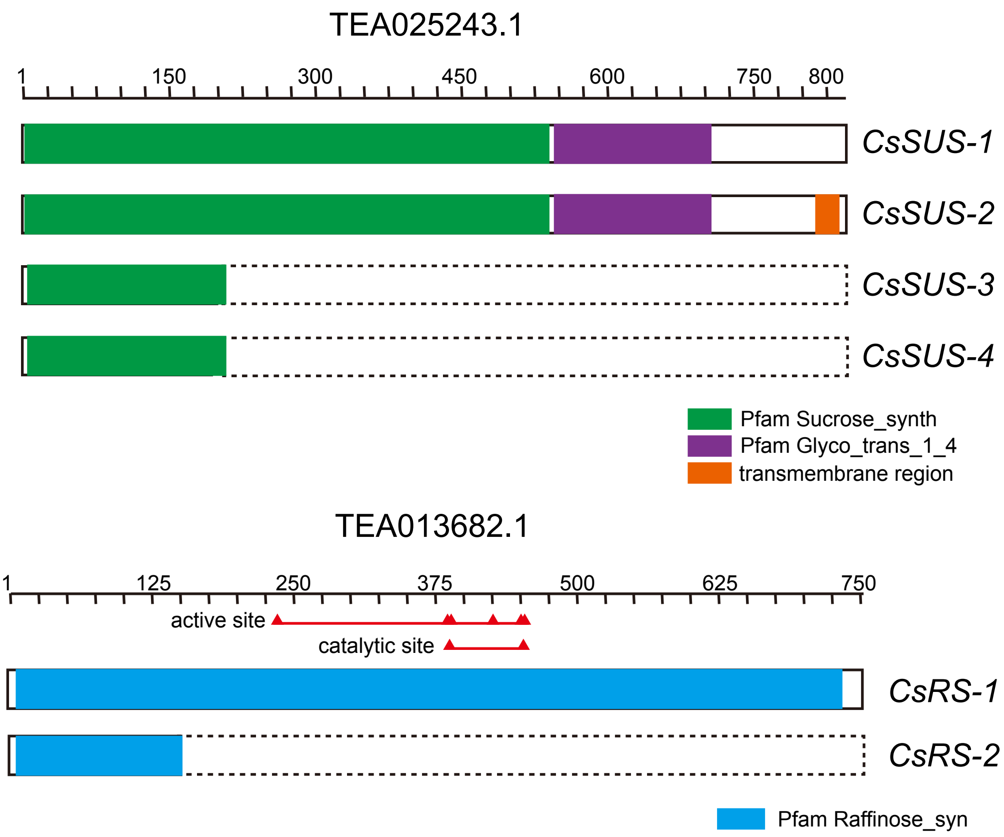

Figure S2. Domain analysis of SUS and RS in AS transcripts. SUS: sucrose synthase; RS: raffinose synthetase.
